# Intravenous IgM-enriched immunoglobulins in critical COVID-19: a multicentre propensity-weighted cohort study

**DOI:** 10.1186/s13054-022-04059-0

**Published:** 2022-07-07

**Authors:** Tim Rahmel, Felix Kraft, Helge Haberl, Ute Achtzehn, Timo Brandenburger, Holger Neb, Dominik Jarczak, Maximilian Dietrich, Harry Magunia, Frieda Zimmer, Jale Basten, Claudia Landgraf, Thea Koch, Kai Zacharowski, Markus A. Weigand, Peter Rosenberger, Roman Ullrich, Patrick Meybohm, Axel Nierhaus, Detlef Kindgen-Milles, Nina Timmesfeld, Michael Adamzik

**Affiliations:** 1 Klinik Für Anästhesiologie, Intensivmedizin Und Schmerztherapie, Universitätsklinikum Knappschaftskrankenhaus Bochum, 44892 Bochum, Germany; 2 Klinische Abteilung Für Allgemeine Anästhesie Und Intensivmedizin, Medizinische Universität Wien, Währinger Gürtel 18-20, 1090 Vienna, Austria; 3 Klinik Für Innere Medizin IV, Klinikum Chemnitz gGmbH, Flemmingstraße 2, 09116 Chemnitz, Germany; 4 Klinik Für Anästhesiologie Und Intensivmedizin, Universitätsklinikum Düsseldorf, Moorenstr. 5, 40225 Düsseldorf, Germany; 5 Klinik Für Anästhesiologie, Intensivmedizin Und Schmerztherapie Universitätsklinikum Frankfurt, Goethe Universität, Theodor-Stern-Kai 7, 60590 Frankfurt am Main, Germany; 6 Klinik Für Intensivmedizin, Universitätsklinikum Hamburg-Eppendorf, Martinistraße 52, 20246 Hamburg, Germany; 7 Klinik Für Anästhesiologie, Universitätsklinikum Heidelberg, Im Neuenheimer Feld 420, 69120 Heidelberg, Germany; 8 Klinik Für Anästhesiologie Und Intensivmedizin, Universitätsklinikum Tübingen, Hoppe-Seyler-Str. 3, 72076 Tübingen, Germany; 9 Abteilung Für Medizinische Informatik, Biometrie Und Epidemiologie, Ruhr-Universität Bochum, Universitätsstraße 105, 44789 Bochum, Germany; 10 Klinik Und Poliklinik Für Anästhesiologie Und Intensivtherapie, Universitätsklinikum Carl Gustav Carus Dresden, Technische Universität Dresden, Fetscherstraße 74, 01307 Dresden, Germany; 11 Abteilung Für Anästhesiologie Und Intensivmedizin, AUVA Traumazentrum Wien, Kundratstraße 37, 1120 Vienna, Austria; 12 Klinik Und Poliklinik Für Anästhesiologie, Intensivmedizin, Notfallmedizin Und Schmerztherapie, Universitätsklinikum Würzburg, Oberdürrbacher Str. 6, 97080 Würzburg, Germany

**Keywords:** Immunoglobulins, Immunoglobulin M, COVID-19, Coronavirus disease, SARS-CoV-2

## Abstract

**Background:**

A profound inflammation-mediated lung injury with long-term acute respiratory distress and high mortality is one of the major complications of critical COVID-19. Immunoglobulin M (IgM)-enriched immunoglobulins seem especially capable of mitigating the inflicted inflammatory harm. However, the efficacy of intravenous IgM-enriched preparations in critically ill patients with COVID-19 is largely unclear.

**Methods:**

In this retrospective multicentric cohort study, 316 patients with laboratory-confirmed critical COVID-19 were treated in ten German and Austrian ICUs between May 2020 and April 2021. The primary outcome was 30-day mortality. Analysis was performed by Cox regression models. Covariate adjustment was performed by propensity score weighting using machine learning-based SuperLearner to overcome the selection bias due to missing randomization. In addition, a subgroup analysis focusing on different treatment regimens and patient characteristics was performed.

**Results:**

Of the 316 ICU patients, 146 received IgM-enriched immunoglobulins and 170 cases did not, which served as controls. There was no survival difference between the two groups in terms of mortality at 30 days in the overall cohort (HR_adj_: 0.83; 95% CI: 0.55 to 1.25; *p* = 0.374). An improved 30-day survival in patients without mechanical ventilation at the time of the immunoglobulin treatment did not reach statistical significance (HR_adj_: 0.23; 95% CI: 0.05 to 1.08; *p* = 0.063). Also, no statistically significant difference was observed in the subgroup when a daily dose of ≥ 15 g and a duration of ≥ 3 days of IgM-enriched immunoglobulins were applied (HR_adj_: 0.65; 95% CI: 0.41 to 1.03; *p* = 0.068).

**Conclusions:**

Although we cannot prove a statistically reliable effect of intravenous IgM-enriched immunoglobulins, the confidence intervals may suggest a clinically relevant effect in certain subgroups. Here, an early administration (i.e. in critically ill but not yet mechanically ventilated COVID-19 patients) and a dose of ≥ 15 g for at least 3 days may confer beneficial effects without concerning safety issues. However, these findings need to be validated in upcoming randomized clinical trials.

*Trial registration* DRKS00025794, German Clinical Trials Register, https://www.drks.de. Registered 6 July 2021.

**Supplementary Information:**

The online version contains supplementary material available at 10.1186/s13054-022-04059-0.

## Background

More than 356 million cases of coronavirus disease 2019 (COVID-19) have been reported worldwide to the end of January 2022, with more than 5.61 million deaths [1]. Until now, only glucocorticoids, interleukin-6 receptor antagonists and janus kinase inhibitors are accepted adjunctive treatments to improve survival among severely ill patients [2]. The benefit from these immunosuppressive substances in critically ill patients corroborates the concept that an exaggerated and prolonged inflammatory response triggers disease progression in COVID-19 [3, 4]. However, there is growing evidence that substantial anti-inflammatory patterns also prevail in critical COVID-19. A profound lymphopenia, for example, is a near uniform finding in critically ill COVID-19 patients, correlating with secondary infections and higher mortality [5]. The assessment of further immunomodulatory approaches seems mandatory since mortality rates in critical COVID-19 remain unacceptably high [6, 7]. Therapeutic options that appreciate both aspects, i.e. inflammatory and anti-inflammatory patterns, embody an especially sophisticated addendum to modulate the harmful immunologic process in critical COVID-19.

Polyvalent immunoglobulin preparations for intravenous use (IVIGs) harbour various immune modulatory properties with the potential of alleviating maladaptive inflammatory and anti-inflammatory patterns in critical COVID-19 [8, 9]. They can stimulate proliferation and restore the repressive function of regulatory T cells, as well as scavenge complement factors and cytokines [10]. Furthermore, IVIGs can limit the proliferation and reactive oxygen species liberation of monocytes and macrophages, counteracting one of the main contributors to severe lung injury in COVID-19 [11, 12]. IVIGs are considered a safe treatment option, rarely associated with serious adverse reactions [13]. Polyvalent immunoglobulin M (IgM) particularly seems to be able to influence the maladaptive immune response in severe COVID-19 as it regulates the activity and survival of the apoptosis inhibitor of macrophages (AIM), thereby improving macrophage survival [14]. Furthermore, IgM is also able to inhibit microvesicle-induced immunothrombosis, one of the main drivers of organ failure in COVID-19 [15]. In this context, IgM-enriched IVIGs (IGAM), which in addition to IgG also contain IgM and IgA, are considered to have a more potent immunomodulatory capacity than conventional IVIG preparations [16] and may represent a promising treatment option in critical COVID-19. However, it seems prudent to identify severely ill patients with COVID-19 who may benefit from IGAM treatment due to the significant costs, the general scarcity of IGAMs and the controversial results of recent studies evaluating conventional IVIG preparations [17–19]. Two randomized controlled trials enrolling moderate to severe COVID-19 ARDS patients to receive IVIG showed no impact on outcome [19, 20], while Gharebaghi et al. [21] presented a small study supporting IVIG treatment to be beneficial for survival. Accordingly, we aimed to assess whether IGAM are (1) Associated with improved 30-day survival, (2) Can enhance faster recovery from organ support and (3) In which patient subgroup an IGAM treatment seems most effective.

## Methods

### Study design, study participants and data collection

This multicentric and multinational retrospective observational study was conducted in ten participating intensive care units (ICUs) in Germany and Austria. This study was approved by the Ethics Committee of the Ruhr-University of Bochum (No. 21-7258) and subsequently by the local ethics committees of each participating centre. The requirement for an individual informed consent was waived due to the retrospective design and the deidentified nature of the data analysed. The study was registered in the “German Register for Clinical Studies” (drks.de: DRKS00025794). Inclusion criteria were an age of ≥ 18 years and a critical course of PCR-confirmed COVID-19 with ICU admission between March 2020 and April 2021. A critical course of COVID-19 was defined according to the World Health Organization as a disease with the confirmation of one (or more) of the following symptoms:

- Respiratory distress, ≥ 30 breaths per minute
- Oxygen saturation ≤ 93% at rest under ambient air or mandatory oxygen
- Oxygenation index ≤ 300 mmHg [arterial oxygen partial pressure (p_a_O_2_)/fractional inspired oxygen (FiO_2_)].
- Presence of respiratory failure, septic shock, and/or multiple organ dysfunction.

### Procedures

Patients enrolled were divided into two groups according to their history of IGAM treatment. The application and indication of IGAM was based on individual indications of the attending physician and compassionate use. The participating centres were instructed to enter at least one control patient with a comparable age and disease severity within the same treatment period for each patient entered in the electronic case report form treated with IGAM. The IGAM group received IgM-enriched IVIGs (consisting of 12% IgM, 12% IgA, and 76% IgG) together with standard care, and the control group was treated with standard care only. In addition, the IGAM subgroup with a high-dose treatment, defined as a total daily dose of IGAMs ≥ 15 g/d and duration of ≥ 3 days, was separated from a lower dose regimen (total daily dose of IGAMs < 15 g/d or duration of < 3 days). Only the total daily dose was considered for group stratification in patients who died within the first three days after starting IGAM treatment, to reduce a potential survivorship bias. All subgroup analyses were performed post hoc.

### Data collection and variables

Each patient’s necessary information was collected from electronic health records of each study centre. All data were entered by each study site in predefined electronic case report forms (REDCap database, version 10.9.4–© 2022 Vanderbilt University) and securely stored at the Ruhr University of Bochum [22]. Each respective local study team first checked and confirmed the validity of the data entered. After the local study teams had verified the data entry, the dataset was additionally validated by an independent intensive care specialist together with a statistician regarding plausibility. Any contradictions were reported as queries and again rechecked and solved by the local study teams as part of the data clearance process.

Clinical and demographic data, including pre-existing comorbidities, age, body mass index, Murray lung injury score, APACHE-II score, sequential organ failure assessment (SOFA) score, need for continuous hemofiltration/dialysis or ECMO therapy, mechanical ventilation settings, pulmonary function, blood chemistry values and outcome, were collected. In this context, the SOFA score was calculated as described by Lambden and colleagues, using the last Glasgow coma score previous to intubation in sedated patients [23]. The supplemental oxygen demand in spontaneously breathing patients was uniformly converted to an FiO_2_ [24]. Data were recorded at ICU admission, the day of the most critical medical condition within the first 10 days after ICU admission, and the day of ICU discharge. Regarding the IGAM group, the day of IGAM treatment initiation and the day of IGAM treatment cessation were also recorded.

### Outcomes

The primary outcome was 30-day survival. Secondary outcomes included the number of ventilator-free days, days without renal replacement therapy and vasopressor-free days, defined as the sum of days without organ support until day 30 after ICU admission. In case of death before day 30, the score for each element was set to − 1. In addition, ventilator-free days and vasopressor-free days were merged as the composite endpoint of organ support-free days. Furthermore, secondary endpoints were ICU-free days and clinical improvement within the first 30 days. The ICU-free days were defined as the number of days between ICU discharge and day 30. All primary and secondary time-to-event outcomes were censored on day 30 as appropriate.

### Statistical analyses

All scores for disease severity were presented as median and interquartile range. All interval-scaled variables which approximately followed a normal distribution were presented as means ± standard deviation (SD) or median and interquartile range (IQR; 25th to 75th percentile), as appropriate. Categorical variables were characterized by numbers with percentage. The confidence intervals (CI) were calculated with 95% coverage. All P values reported are nominal and two-sided with an a priori significance level of less than 0.05.

Kaplan–Meier curves, log-rank tests and Cox regression models were calculated for primary and secondary outcomes as appropriate. Results of Cox regression models are presented using hazard ratios (HRs). We assumed that groups were not comparable without balancing control and IGAM patient’s characteristics due to the missing randomization and, thus, the assumption of an inherent selection bias. Therefore, baseline characteristics, such as age, gender, body mass index and admissions status, underlying diseases, long-term medications, disease severity, relevant laboratory parameters and adjuvant COVID-19 therapies, were surveyed. We fitted Cox regression models within each hospital including the different participating hospitals as strata because baseline hazards also varied across participating centres according to the retrospective study design. Therefore, separate baseline hazards for each strata were estimated. We estimated propensity scores using the SuperLearner algorithm [25] and then converted those propensity scores into weights using a formula that depends on the desired estimate to attain balance between all covariates (Additional file 1 and Additional file 2). We employed the following models in our SuperLearner algorithm: LASSO Breiman Random Forest, eXtreme Gradient Boosting (Random Forest) and Bayesian Additive Regression Trees, as described previously, for a maximum accuracy [26]. Furthermore, we added the simple mean as a benchmark algorithm and simple logistic and logistic regression with interaction terms to our SuperLearner approach. All statistical analyses were performed using R version 4.1.2.

### Role of funding sources

Study costs were covered primarily from institutional and/or departmental sources of all participating study centres. Furthermore, Biotest AG partially covered administrative study costs (i.e. costs for database management and statistical analysis), without having any impact on the study design, study conduct, data collection, data analysis, data interpretation, publication of results or other relevant aspects. Only study sites and the group of investigators who participated in the trial are responsible for the entire scientific content.

## Results

### Patient characteristics

A total of 316 patients with critical COVID-19 were enrolled in our study and included in the data analysis. The IGAM group consisted of 146 (46%) patients and the control group comprised 170 (54%) patients. Baseline characteristics were typical for a population with critical COVID-19, but not in all aspects (including disease severity) balanced between the IGAM and the control group (Table 1). The patients in the IGAM group were younger at 59.4 ± 12.7 years compared to 62.5 ± 11.6 years (*p* = 0.027) in the control group. Patients in the IGAM group also suffered more frequently from malignancies 23.3% (34 of 146) and had a lower body mass index 30.2 ± 5.8 kg/m^2^ compared to 8.2% (14 of 170; *p* < 0.001) and 31.9 ± 7.2 kg/m^2^ (*p* = 0.020) in the control group. In addition, 12.3% (18 of 146) in the IGAM group and only 4.1% (7 of 170) of the control group received immunosuppressive agents as long-term medication (*p* = 0.013).

**Table 1:** Baseline characteristics of study population (*n* = 316)

	IGAM group (*n* = 146)	Control group (*n* = 170)	*p* value
*Demographics*			
Age [years], mean (± SD)	59.4 (± 12.7)	62.5 (± 11.6)	**0.027**
Female sex, n (%)	36 (24.7%)	43 (25.3%)	1.000
Body mass index [kg/m^2^], mean (± SD)	30.2 (± 5.8)	31.9 (± 7.2)	**0.020**
*Comorbidities, n (%)*			
None	26 (17.8%)	26 (15.3%)	0.654
Hypertension	60 (41.1%)	55 (32.4%)	0.135
Cardiovascular disease	37 (26.9%)	48 (28.2%)	0.652
Chronic heart failure	18 (12.3%)	13 (7.7%)	0.228
Chronic kidney disease	22 (15.1%)	17 (10.0%)	0.232
Chronic obstructive pulmonary disease	13 (8.9%)	10 (5.9%)	0.416
Diabetes mellitus	41 (28.1%)	60 (35.3%)	0.211
Malignant disease	34 (23.3%)	14 (8.2%)	** < 0.001**
*Permanent medication, n (%)*			
None	41 (28.1%)	51 (30.0%)	0.803
ACEI	31 (21.2%)	46 (27.1%)	0.284
ARB´s	18 (12.3%)	33 (19.4%)	0.120
Beta blockers	52 (35.6%)	54 (31.8%)	0.546
Platelet aggregation inhibitors	33 (22.6%)	43 (25.3%)	0.670
Anticoagulants	13 (8.9%)	18 (10.6%)	0.755
Corticosteroids	21 (14.4%)	18 (10.6%)	0.395
Immunosuppressive agents	18 (12.3%)	7 (4.1%)	**0.013**
Polypharmacy (≥ 5 drugs)	53 (36.3%)	71 (41.8%)	0.381
*Status at ICU admission*			
Time between symptom onset and ICU admission, median (IQR)	9 [5, 14]	9 [6, 14]	0.986
Respiratory Support			0.711
Supplemental oxygen, n(%)	23 (15.8%)	24 (14.1%)	
High-flow oxygen device, n(%)	25 (17.1%)	38 (22.4%)	
Non-invasive ventilation, n(%)	21 (14.4%)	23 (13.5%)	
Mechanical ventilation, n(%)	77 (52.7%)	85 (50%)	
APACHE-II score	18.0 (± 8.7)	16.9 (8,1)	0.233
SARS-CoV-2 virus load [CT value]; mean (± SD)	26.5 (± 5.9)	28.4 (6.31)	0.072
COVID-19 course—day with highest disease severity*			
Days after ICU admission [days], median (IQR)	4 [1; 8]	3 [1; 6]	**0.015**
Respiratory Support			0.294
Supplemental oxygen, n(%)	3 (2.1%)	3 (1.8%)	
High-flow oxygen device, n(%)	6 (4.1%)	15 (8.8%)	
Non-invasive ventilation, n(%)	13 (8.9%)	10 (5.9%)	
Mechanical ventilation, n(%)	124 (84.9%)	142 (83.5%)	
Horowitz index [PaO2/FiO2], median (IQR)	88 [69; 139]	105 [75; 163]	**0.011**
P_insp_ [cmH_2_O], median (IQR)	27 [23; 30]	27 [23; 30]	0.635
PEEP [cmH_2_O], median (IQR)	12 [10; 14]	12 [10; 14]	0.327
Murray score; median (IQR)	13 [10; 14]	12 [9; 14]	**0.027**
SOFA score; median (IQR)	10 [8; 13]	9 [7; 12]	**0.022**
AKI KDIGO stage, n (%)			0.335
No acute renal injury	73 (50.0%)	100 (58.8)	
1	17 (11.6%)	21 (12.4%)	
2	8 (5.5%)	6 (3.5%)	
3	48 (32.9%)	43 (25.3%)	
Vasopressor support, n (%)	119 (81.5%)	129 (75.9%)	
SARS-CoV-2 virus load [CT value]; mean (± SD)	24.6 (± 7.0)	30.1 (± 6.0)	** < 0.001**
Laboratory values—of day with highest disease severity*; median (IQR)			
Leukocyte count [1000/µL]	11.9 [8.0; 20.1]	11.7 [8.4; 16.2]	0.760
Neutrophile count [1000/µL]	9.1 [6.3; 14.6]	9.6 [6.5; 13.8]	0.688
Lymphocyte count [1000/µL]	0.9 [0.5; 1.5]	0.9 [0.5; 1.4]	0.907
C-reactive protein [mg/L]	153 [113; 262]	149 [83; 206]	0.082
Procalcitonin [ng/mL]	0.9 [0.3; 3.0]	0.5 [0.2; 1.2]	**0.001**
Interleukin-6 [pg/mL]	247 [82; 741]	139 [60; 376]	**0.038**
Ferritin [µg/L]	1638 [935; 4261]	1304 [668; 2237]	**0.004**
Platelet count [1000/µL]	179 [116; 281]	246 [158; 336]	**0.001**
Serum creatinine [mg/dL]	1.13 [0.74; 1.63]	0.93 [0.69; 1.56]	0.162
D-dimers [µg/mL]	2.67 [1.41; 7.1]	2.37 [1.2; 4.62]	0.199
Total bilirubin [mg/dL]	0.8 [0.5; 1.8]	0.6 [0.4; 1.1]	**0.009**
IgM serum concentration [mg/dL]	67 [29; 122]	80 [63; 115]	**0.052**
IgA serum concentration [mg/dL]	200 [148; 282]	248 [189; 327]	0.089
IgG serum concentration [mg/dL]	772 [553; 1060]	916 [784; 1063]	**0.048**
*Adjunctive therapies; n (%)*			
Corticosteroids	125 (85.6%)	139 (81.8%)	0.442
Interleukin-6 receptor antagonist	12 (8.22%)	22 (12.9%)	0.243
Remdesivir	22 (15.1%)	36 (21.2%)	0.210
*IGAM treatment characteristics; median (IQR)*			
Initiation time [days after ICU admission]	4 [1; 11]	n/a	
Treatment: duration [days]	3 [3; 4]	n/a	
Treatment: daily dose [g]	23.2 [17.6; 25.8]	n/a	

Statistically significant *p*-values are presented in bold

*ACEI* angiotensin converting-enzyme inhibitor; *ARB* angiotensin II receptor blocker; *ICU* intensive care unit; *APACHE-II score* acute physiology and chronic health evaluation II score; *CT* cycle threshold; *PaO*_*2*_ partial pressure of oxygen; *FiO*_*2*_ fraction of inspired oxygen; *PEEP* positive end expiratory pressure; *P*_*insp*_ inspiratory plateau pressure; *SOFA score* sequential organ failure assessment score; *AKI* acute kidney injury; *KDIGO* kidney disease: improving global outcomes; *IGAM* IgM-enriched intravenous immunoglobulins

*day of the most critical medical condition within the first 10 days after ICU admission

The patients were admitted to the ICU in a median of 9 days (IQR: 5 to 14 days) after the onset of the first symptoms in the IGAM group and 9 days (IQR: 6 to 14 days) in the control group (*p* = 0.986). At that time point, all patients needed respiratory support. In detail, 14.9% of the total cohort (47 of 316) received supplemental oxygen, 19.9% (63 of 316) high-flow nasal cannula, 13.9% (44 of 316) non-invasive ventilation, and 51.3% (162 of 316) invasive mechanical ventilation at ICU admission, without any difference between the IGAM and control group (*p* = 0.711, Table 1). However, patients in the IGAM group exhibited a higher disease severity within the first 10 days, as reflected in a higher SOFA score of 10 (IQR: 8 to 13), a higher Murray lung injury score of 13 (IQR: 10 to 14) and a lower oxygenation index of 88 mmHg (IQR: 69 to 139 mmHg) compared to 9 (IQR: 7 to 12; *p* = 0.022), 12 (IQR: 9 to 14; *p* = 0.027) and 105 mmHg (IQR: 75 to 163 mmHg, *p* = 0.011) in the control group, respectively. This was also underpinned by respective laboratory markers, i.e. a higher virus load and higher serum concentrations of total bilirubin, procalcitonin, interleukin-6 and serum ferritin in the IGAM group (Table 1). Baseline characteristics of the investigated subgroups are provided as Additional file 3.

The majority of patients were enrolled after the announcement of the effect of dexamethasone in the RECOVERY trial [7]. Therefore, 83.5% (264 of 316) of all patients received dexamethasone as an adjunctive therapy in our cohort, with no difference between the IGAM and control group (85.6% vs. 81.8%; *p* = 0.442).

### Primary outcome

**Fig. 1:**
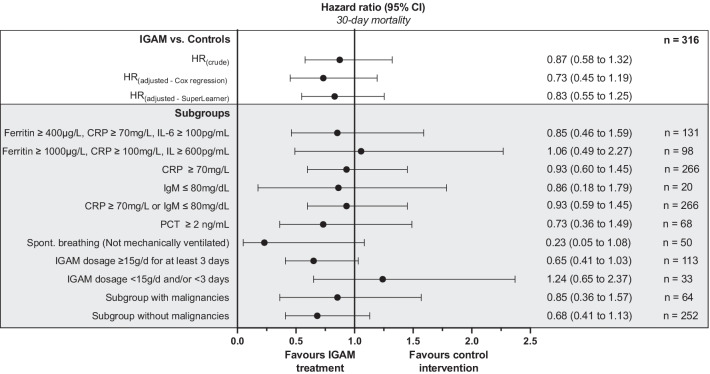
Adjusted hazard ratios of COVID-19 patients regarding 30-day survival. Intergroup imbalances were adjusted using multivariate Cox regression and different propensity score weighting approaches. The rows represent different groups/subgroup analysis of COVID-19 patients

The non-adjusted 30-day mortality in the IGAM group was 28.8% (42 of 146) compared to 31.8% (54 of 170) in the control group. An adjusted impact on 30-day survival was assessed by propensity score weighting using the machine learning-based SuperLearner because several baseline characteristics were not balanced across the IGAM and the control group. The adjusted hazard ratio (HR_adj_) of 0.83 (95% CI: 0.55 to 1.25; *p* = 0.374) did not indicate any impact on 30-day survival in dependent of an IGAM treatment (Fig. 1).

A subgroup analysis of patients with a high-dose IGAM treatment showed an adjusted hazard risk of 0.65 (95% CI: 0.41 to 1.03; *p* = 0.068; Fig. 1) for the IGAM group regarding 30-day mortality. We assessed the subgroup of not yet mechanically ventilated patients separately to explore the impact of an appropriate IGAM application timing. In this subgroup, we found a HR_adj_ of 0.23 regarding 30-day mortality, however, without reaching statistical significance (95% CI: 0.05 to 1.08; *p* = 0.063; Fig. 1). This observation was corroborated by the finding that an IGAM administration < 14 days after the onset of symptoms also exhibited an adjusted hazard risk of 0.62 (95% CI: 0.34 to 1.10; *p* = 0.100) regarding 30-day mortality. The 30-day mortality in a subgroup excluding patients with malignant diseases as an important confounder tended to be lower in the IGAM group compared to controls (HR_adj_: 0.68; 95% CI: 0.41 to 1.13; *p* = 0.136). None of the other subgroups investigated affirmed an impact on 30-day mortality independent of an IGAM treatment (Fig. 1).

### Secondary outcomes

Secondary outcomes are listed in Table 2. The median number of organ support-free days was 0 [IQR: -1; 10] in the IGAM group and 0 [IQR: − 1; 22] in the control group. The adjusted HR (HR_adj_) using propensity score weighted SuperLearner regarding organ support-free days was 1.03 (95% CI: 0.81 to 1.32, *p* = 0.817) for the IGAM group compared with the control group, not suggesting whether there was any adverse or beneficial impact on this endpoint. Similarly, the number of ventilator-free days on day 30 (HR_adj_: 1.10; 95% CI: 0.87 to 1.38; *p* = 0.443) and vasopressor-free days on day 30 (HR_adj_: 1.00; 95% CI: 0.79 to 1.26; *p* = 0.994) were no different between the groups. In addition, the number of dialysis-free days was comparable in the IGAM 9 [IQR:− 1; 19] and control group 8.5 [IQR: − 1; 19] (HR_adj_: 1.03; 95% CI: 0.79 to 1.34; *p* = 0.828).

**Table 2:** Clinical outcome variables (adjusted using SuperLearner)

	IGAM group (*n* = 146)	Controls (*n* = 170)	HR_adjusted_ (95%-CI)	*p value*
*Primary outcome*				
30-day mortality, n (%)	42 (28.8%)	54 (31.8%)	0.83 (0.55 to 1.25)	0.374
*Secondary outcomes, median [IQR]*				
Organ support-free days at 30 days	0 [-1; 10]	0 [-1; 22]	1.03 (0.81 to 1.32)	0.817
Ventilator-free days at 30 days	0 [-1; 16]	1 [-1; 22]	1.10 (0.87 to 1.38)	0.443
Vasopressor-free days at 30 days	0 [-1; 21.5]	1.5 [-1; 29]	1.00 (0.79 to 1.26)	0.994
Dialysis-free days at 30 days	9 [-1; 19]	8.5 [-1; 19]	1.03 (0.79 to 1.34)	0.828
ICU length of stay	24 [14; 40]	20 [10; 34]	n/a	0.040
ICU-free days at 30 days	0 [-1; 4]	0 [-1; 6]	n/a	0.943
Hospital length of stay	33 [20; 50]	25.5 [16; 42]	n/a	0.010

*IGAM* IgM-enriched intravenous immunoglobulins; *ICU* intensive care unit

Furthermore, there was no treatment-dependent pattern between IGAM patients and controls regarding the incidence of secondary bacterial infections (76.0 vs. 71.8%, respectively; *p* = 0.465).

## Discussion

We found in this retrospective propensity-weighted cohort study of critically ill COVID-19 patients that IGAM treatment may confer beneficial effects in certain subgroups. In our cohort, patients not yet mechanically ventilated and those receiving ≥ 15 g daily for at least 3 days were most likely to benefit from an IGAM treatment, although we could not reach statistically significant differences compared to controls. Therefore, these findings must be tested for plausibility and causality in upcoming randomized trials.

The use of IVIGs in COVID-19 was initially reported by Cao et al. [17] who described a remarkable benefit in three deteriorating patients. Most of the studies published subsequently also reported a favourable clinical response, confirmed by the resolution of lung lesions with a normalization of oxygen saturation and global improvement in clinical status [18, 27, 28]. Gharebaghi et al. [21] in one of the first randomized placebo-controlled double-blind clinical trials confirmed the IVIGs were independently associated with a lower in-hospital mortality. Furthermore, a recent meta-analysis performed by Xiang and colleagues confirmed the clinical efficacy of IVIGs by showing a favourable impact on survival in critically ill COVID-19 patients [28]. Nevertheless, the existing literature on IVIGs in COVID-19 is still controversial, which can be mainly explained by the high degree of heterogeneity in disease severity, different disease stages, small cohorts and a lack of standardized treatment regimens across the different studies [19, 29]. In this context, a large randomized controlled trial could not demonstrate any benefit on outcome, but even a trend towards an increased frequency of serious adverse events [19].

This problem might be solved by selecting the right patients, in the sense of personalized medicine. A recent large multicentre retrospective cohort study reported that treatment with IVIG for at least 5 days led to a significant decrease in 28-day mortality in critically ill COVID-19 patients [18]. The study also elucidated that beneficial effects of an IVIG treatment were more pronounced in appreciation of the right timing, i.e. ≤ 7 days from ICU admission [18]. Strikingly, further studies also found an association between treatment efficiency and a sufficient dosage and appropriate timing. Xie et al. [30], for example, reported the highest reduction in 28-day mortality in the case of IVIG administration within 48 h of ICU admission. This is consistent with our two results, indicating a trend towards relevant treatment effects in both patients who had not yet received mechanical ventilation, or those for whom IGAM treatment was not initiated until day 14 after the onset of first symptoms, respectively. This fact becomes particularly important because several other studies focused exclusively on patients already receiving mechanical ventilation at the start of IVIG treatment, thus focusing on later stages of the disease. In those studies, no clear evidence exists that IVIGs were effective in preventing disease progression or having a beneficial impact on survival [19, 20, 29]. In particular, a large randomized controlled trial from France showed no improved clinical outcome at day 28 by using IVIGs in patients already receiving mechanical ventilation [19]. These findings are in line with our study results, as we could not demonstrate a beneficial effect for IGAMs in patients receiving mechanical ventilation at treatment initiation or being treated with IGAMs after day 14 (HR: 0.93; 95% CI: 0.58 to 1.52; *p* = 0.791). Therefore, it seems crucial to pay attention to a timely appropriate administration of IVIGs. Here, the recent studies and our results indicate that administration before mechanical ventilation is required, in order to achieve the most beneficial effects.

Furthermore, data on mortality among different studies are not conclusive, due to different IVIG dosing regimens, preparations and different IVIG treatment durations. The recommended dose of IgM-enriched preparations in patients with hyperinflammation is 0.25 g/kg per day for at least 3 days [8]. However, the daily doses administered in our multicentric study differed between patients, ranging from 0.05 to 0.4 g/kg. In addition, the duration of the treatment also varied from 1 to 7 days. A subgroup analysis revealed that a dose of ≥ 15 g per day, equivalent to a daily dose of 0.2 to 0.3 g/kg, for at least 3 days may be superior to lower dose regimens, in line with previous studies in sepsis and COVID-19 [18, 31]. It is noteworthy that our results do not imply a definitive dosage or treatment recommendation. However, our results may indicate a dosage threshold at which favourable treatment possibly occur. Bearing these considerations in mind, a solidifying picture emerges supporting IGAM in certain subgroups of critically ill COVID-19 patients. However, this preliminary picture needs to be tested for causality in a prospective randomized controlled setting prior to implementation in clinical practice.

The clinical difficulties in treating this new disease also arise from the fact that we cannot reliably discriminate distinctive disease stages and different patients’ phenotypes, although it seems important to administer IGAM at the right time. Several clinical or biological markers, including ferritin, C-reactive protein, and pro-inflammatory interleukin-1 and interleukin-6, have currently been identified to help predict the course of COVID-19 and, therefore, can potentially help to guide IGAM usage [32]. However, as shown in our subgroup analysis, alteration of these inflammatory markers does not appear as suitable or rather timely biomarkers in COVID-19 to indicate appropriate initiation of treatment with IGAM. In this context, we hypothesize that when our applied thresholds for inflammatory markers were reached, the inflammatory process was likely too advanced and the therapeutic window for a sufficient IGAM therapy was already closed. Thus, our data suggest that the decision regarding the initiation of IGAM treatment should currently be based on clinical parameters (i.e. not yet mechanically ventilated and/or within the first 14 days after the onset of symptoms) rather than on inflammatory biomarkers.

Of course, it is also necessary to discuss the question of the potential advantages of IGAM treatment compared to established immunotherapies. As inflammatory properties are considered the pathogenic basis for disease progression in critical COVID-19, most of the approved agents show anti-inflammatory properties [3]. Although silencing the inflammation is currently the most favoured solution, there is growing evidence that profound anti-inflammatory patterns also prevail in critical COVID-19 [33]. Therefore, it seems prudent when adjunctive therapies are also capable of tackling multiple immunologic perturbations in addition to inflammation. In more concrete terms, adjunctive therapies in COVID-19 should, firstly, control and resolve the prolonged inflammation, secondly, augment the restoration from immune dysregulation and, thirdly, should not harm, for example, due to the susceptibility of the host to secondary infections. In this sense, IVIGs may be capable of modulating the activity of the cytokine network, neutralizing autoantibodies, and regulating the proliferation and differentiation of immune cells [10]. Therefore, especially IGAMs may represent promising candidates because they act on all three levels with synergistic mechanisms, helping to restore immune homeostasis [34].

Although the peaking inflammatory phase of COVID-19 progression is often imprecisely described as a “cytokine storm” [35], recent studies have shown that systemic levels of cytokines may not be as high as seen in ‘classical’ sepsis or acute respiratory distress syndrome [33, 36]. In this regard, we also detected, even on the day with the most critical medical condition, only moderately elevated concentrations of interleukin-6, serum ferritin and C-reactive protein. In line with a growing number of recently published studies describing this issue, the concentrations of inflammatory mediators measured do not fit into the classic picture of excessive hyperinflammation [33, 36]. By now, the immunological picture of severe COVID-19 has evolved to a moderate but persistent inflammation maintained by macrophages, which also helps to explain the efficacy of IGAM in COVID-19. The IgM via the protein AIM plays an especially vital role in macrophage activity and homeostasis [14, 37]. Given the important role of macrophages in SARS-CoV-2-induced immune responses, targeted reprogramming of macrophages via IgM substitution to stabilize anti-inflammatory M2 phenotypes by the scavenging of AIM may indicate a promising approach [38]. Additionally, IGAMs also target several other important immunological pathways in COVID-19, such as inhibiting microvesicle-driven immunothrombosis [15]. Thus, IGAM may have substantial advantages over classical IVIGs as an adjunctive treatment in critical COVID-19 [16, 39]. However, these mechanistical considerations need to be explored in future experimental studies.

Taken together, despite the huge clinical heterogeneity, we were able to reveal that an early and high-dose treatment may improve the prognosis of critically ill COVID-19 patients. However, neither the early use of IGAM (i.e. in not yet mechanically ventilated patients) nor a sustained high-dose (i.e. ≥ 15 g/d for at least 3 days) was associated with a markedly improved number of organ support-free days. Still, we see the possibility that IGAM can attenuate the burden of the critical COVID-19 disease and should be urgently evaluated for effectiveness in upcoming clinical trials.

### Limitations

All results are of an associative nature due to the retrospective design of our study. In addition, all subgroup analyses arose post hoc, and thus conclusions derived from these results must be interpreted carefully. Therefore, no direct treatment recommendations should be derived from our results, despite several strengths, such as the number of cases and the multicentric character. Secondly, the selection of the primary endpoint can also be critically discussed, as we did not assess long-term effects may occur from the chosen study design. However, IGAM treatment may not necessarily impact the underlying cause of death at later time points because patients with COVID-19 beyond day 30 also face multiple other risks. Thirdly, despite the greatest statistical effort to control confounders, we cannot entirely exclude an existing bias, even after confirming our results in different approaches to adjust for intergroup imbalance (i.e. SuperLearner, inverse probability of treatment weighting, Random-Forest and multivariate proportional hazards Cox regression; see Additional file 1). Nevertheless, residual confounding may exist, e.g. due to immortal time bias, which could limit the reproducibility of the data. Fourthly, our results may be affected by different patterns of physician’s treatment practice over time. Finally, we cannot entirely clarify to what extent the concurrent use of glucocorticoids or adjunctive drugs, such as Remdesivir, Tocilizumab or Anakinra, may impact the effect of IGAM treatment. Therefore, the need for a combined use of IGAM in future work should also be explored.

## Conclusions

Our confidence intervals suggest potentially favourable treatment effects when using IGAM in critically ill but not yet mechanically ventilated COVID-19 patients and in patients receiving a dose of ≥ 15 g for at least 3 days. Both factors may be necessary to achieve clinically relevant treatment effects in critically ill COVID-19 patients. In addition, our data indicate that the usage of IGAM is safe and not associated with fatal adverse events. Therefore, our study provides crucial clinical insights which should be reflected in the design of upcoming randomized controlled trials.

## Supplementary Information

**Additional file 1** Additional results on primary outcome (30-day mortality) by using different statistical methods for bias control.

**Additional file 2** Covariates included in the models.

**Additional file 3** Baseline characteristics of investigated subgroups.

## Data Availability

Data can be obtained from the authors upon reasonable request.

## Abbreviations

AIM: Apoptosis inhibitor of macrophages
CI: Confidence intervals
COVID-19: Coronavirus disease 2019
ECMO: Extracorporeal membrane oxygenation
F_i_O_2_: Fractional inspired oxygen
HR: Hazard ratio
ICU: Intensive care unit
IGAM: Polyvalent IgM-enriched immunoglobulin preparations for intravenous use
IgA: Immunoglobulin A
IgG: Immunoglobulin G
IgM: Immunoglobulin M
IQR: Interquartile range
IVIG: Polyvalent immunoglobulin preparations for intravenous use
PCR: Polymerase chain reaction
p_a_O_2_: Arterial oxygen partial pressure
SD: Standard deviation
SOFA score: Sequential organ failure assessment score
