# Supplementary material for: Intravenous IgM-enriched immunoglobulins in critical COVID-19: a multicentre propensity-weighted cohort study
Source: Crit Care. 2022 Jul 7;26:204. doi: 10.1186/s13054-022-04059-0 (PMC9260992; doi:10.1186/s13054-022-04059-0)
Supplement: Supplementary file 1 — Additional file 1 Additional results on primary outcome (30-day mortality) by using different statistical methods for bias control. [file 13054_2022_4059_MOESM1_ESM.pdf]

<u>Additional File 1:</u>

Additional results on primary outcome (30-day mortality) by using different statistical methods for bias control

Explanation of different Cox models:

Crude: Cox model with treatment and centres as strata but without any adjustment of covariates

p-value based selection using values at baseline, the first ten days and differences: stepwise variable selection procedure (with iterations between the 'forward' and 'backward' steps) using p-value based variable inclusion and exclusion to obtain the best candidate final Cox's proportional hazards model or Cox's proportional hazards model with time-dependent covariates. Cut-off for inclusion/exclusion was 0.1. After model selection, the treatment variable and the centers as strata were added to the model to study the treatment effect.

Liberal: multivariate Cox model, taking all variables into account that have a significant influence on survival at the 5% level and were collected from at least 90% of the patients.

Restrictive: Cox model including the following covariates: treatment indicator, age, referral status, underlying malignancies, use of immunosuppressants, polypharmacy indicator (patient is considered a polypharmacy patient if he/she is taking at least 5 preparations in his/her long-term medication), SOFA score on the worst day within the first 10 days in the ICU. Covariate selection due to significant impact on outcome and heterogenous ($p<0.05$) distribution between IGAM and control group.

SuperLearner: method relies on estimating propensity scores using the SuperLearner algorithm and then converting those propensity scores into weights using a formula that depends on the desired estimand. SuperLearner is an algorithm that uses cross-validation to estimate the performance of multiple machine learning models, or the same model with different settings. It then creates an optimal weighted average of those models, aka an "ensemble", using the test data performance. This approach has been proven to be asymptotically as accurate as the best possible prediction algorithm that is tested. For maximum accuracy, the Guide to SuperLearner (https://cran.r-project.org/web/packages/SuperLearner/vignettes/Guide-to-SuperLearner.html) recommends trying at least the following models: glmnet (LASSO regression), randomForest (Breiman Random Forest), XGBoost (eXtreme Gradient Boosting), and bartMachine (Bayesian Additive Regression Trees). Furthermore, we add: mean (simple mean), glm (simple logistic regression) and glm.interaction (logistic regression with interaction terms).

Balanced SuperLearner (BalSL): Using Balance SuperLearner by setting SL.method = "method.balance" works by selecting the combination of predicted values that minimizes an imbalance measure (only for binary treatment).

Generalized Boosted Models (GBM): method relies on estimating propensity scores using generalized boosted modeling and then converting those propensity scores into weights using a formula that depends on the desired estimand. Generalized boosted modeling (GBM, also known as gradient boosting machines)

is a machine learning method that generates predicted values from a flexible regression of the treatment on the covariates, which are treated as propensity scores and used to compute weights. It does this by building a series of regression trees, each fit to the residuals of the last, minimizing a loss function that depends on the distribution chosen.

Subgroup Balancing Propensity Score (SBPS) with LASSO regression/SuperLearner: algorithm that attempts to achieve balance in subgroups by sharing information from the overall sample and subgroups (here: centers (`redcap_data_access_group`) as subgroups) (Dong, Zhang, Zeng, & Li, 2020; DZZL). Each subgroup can use either weights estimated using the whole sample, weights estimated using just that subgroup, or a combination of the two. The optimal combination is chosen as that which minimizes an imbalance criterion that includes subgroup as well as overall balance

Total population

Control (n= 170) vs. IGAM (n=146)

Black font: including centres as additional strata, *red font:* not including centres as additional strata

Cox model	HR	CI	p-value	AIC	# events	# observations
Crude	0.873<br>0.810	(0.577 -- 1.321)<br>(0.541 -- 1.213)	0.520<br>0.306	640.722<br>1055.474	96	316
p-value based selection using values at baseline, the first ten days and differences	0.735<br>0.706	(0.456 -- 1.187)<br>(0.453 – 1.099)	0.208<br>0.123	515.398<br>865.548	85	285
Liberal	0.733<br>0.683	(0.450 -- 1.193)<br>(0.432 -- 1.079)	0.211<br>0.102	532.924<br>879.260	85	284
Restrictive	0.698<br>0.727	(0.449 -- 1.084)<br>(0.475 -- 1.112)	0.110<br>0.142	608.411<br>1025.331	96	316
SuperLearner (SL)	0.830<br>0.779	(0.549 -- 1.253)<br>(0.519 -- 1.168)	0.374<br>0.227	851.911<br>1382.559	96	316
Balanced SuperLearner (BalSL)	0.802<br>0.747	(0.528 -- 1.218)<br>(0.495 – 1.129)	0.300<br>0.166	1000.973<br>1582.493	96	316
GBM	0.719<br>0.707	(0.470 -- 1.101)<br>(0.466 – 1.073)	0.129<br>0.103	1070.416<br>1679.836	96	316
SBPS with LASSO regression	0.818	(0.528 -- 1.267)	0.369	1205.101	86	283
SBPS with SuperLearner	0.809	(0.524 -- 1.247)	0.336	116.066	86	283

Control vs. high-dose Pentaglobin (>15/d for at least three days)

Control (n= 170) vs. IGAM (n=113)

Black font: Adjustment without centre as strata due to sample size.

**Cox model**	**HR**	**CI**	**p-value**	**AIC**	**# events**	**# observations**
Crude	0.723	(0.458 -- 1.142)	0.164	1056.143	96	283
p-value based selection using values at baseline, the first ten days and differences	0.638	(0.390 -- 1.044)	0.074	866.530	85	283
Liberal	0.607	(0.363 -- 1.016)	0.057	880.167	85	283
Restrictive	0.667	(0.414 – 1.076)	0.097	1026.638	96	283
SuperLearner (SL)	0.653	(0.413 -- 1.032)	0.068	1994.309	96	283
GBM	0.642	(0.399 -- 1.032)	0.067	1679.836	96	283

Control vs. high-dose Pentaglobin (>0.25g/kg bodyweight for at least three days)

Control (n= 170) vs. IGAM (n=88)

Black font: Adjustment without centre as strata due to sample size.

**Cox model**	**HR**	**CI**	**p-value**	**AIC**	**# events**	**# observations**
Crude	0.709	(0.381 -- 1.320)	0.164	1056.143	96	258
p-value based selection using values at baseline, the first ten days and differences	0.568	(0.297 -- 1.085)	0.087	609.596	96	258
Liberal	0.540	(0.250 -- 1.163)	0.116	533.782	85	258
Restrictive	0.589	(0.284– 1.211)	0.155	516.711	85	258
SuperLearner (SL)	0.577	(0.309 -- 1.077)	0.074	1075.520	96	258
GBM	0.437	(0.218 – 0.879)	0.020	1408.265	96	258

Subgroups - Not mechanically ventilated

Control (n= 28) vs. IGAM (n=22)

Black font: Adjustment without centre as strata due to sample size.

Cox model	HR	CI	p-value	AIC	# events	# observations
Crude	0.235	(0.047 -- 1.183)	0.079	54.459	8	50
p-value based selection using values at baseline, the first ten days and differences	0.232	(0.043 -- 1.243)	0.088	57.210	8	50
Liberal	0.073	(0.007 -- 0.722)	0.025	41.667	7	47
Restrictive	0.096	(0.008 -- 1.161)	0.065	57.382	8	50
SuperLearner (SL)	0.230	(0.049 -- 1.083)	0.063	85.587	8	50
GBM	0.237	(0.049 -- 1.148)	0.074	94.151	8	50

Subgroups - Ferritin ≥ 400μg/l, CRP ≥ 70mg/l, IL-6 ≥100pg/ml (n=131)

Black font: Adjustment without centre as strata due to sample size.

Cox model	HR	CI	p-value	AIC	# events	# observations
Crude	0.859	(0.462 -- 1.597)	0.631	372.645	40	131
p-value based selection using values at baseline, the first ten days and differences	1.245	(0.616 -- 2.515)	0.542	323.594	38	118
Liberal	1.104	(0.461 -- 2.644)	0.825	259.491	32	110
Restrictive	0.889	(0.450 -- 1.756)	0.735	360.148	40	131
SuperLearner (SL)	0.853	(0.459 -- 1.585)	0.616	715.840	40	131
GBM	0.780	(0.415 -- 1.465)	0.440	630.782	40	131

Subgroups - Ferritin ≥ 1000μg/l, CRP ≥ 100mg/l, IL-6 ≥600pg/ml (n=98)

Black font: Adjustment without centre as strata due to sample size.

Cox model	HR	CI	p-value	AIC	# events	# observations
Crude	0.740	(0.381 -- 1.437)	0.374	305.091	35	98
p-value based selection using values at baseline, the first ten days and differences	0.898	(0.442 -- 1.822)	0.766	300.338	35	98
Liberal	1.375	(0.663 -- 2.853)	0.393	240.829	31	86
Restrictive	0.740	(0.382 -- 1.434)	0.373	294.765	35	98
SuperLearner (SL)	1.056	(0.491 -- 2.268)	0.889	666.150	35	98
GBM	0.679	(0.345 -- 1.334)	0.261	519.182	35	98

Subgroups -* CRP ≥ 70mg/l *(n=266)

Black font: Adjustment without centre as strata due to sample size.

Cox model	HR	CI	p-value	AIC	# events	# observations
Crude	1.000	(0.643 -- 1.555)	0.999	848.343	79	266
p-value based selection using values at baseline, the first ten days and differences	0.714	(0.415 -- 1.227)	0.223	593.602	61	206
Liberal	0.753	(0.443 -- 1.281)	0.295	733.091	71	244
Restrictive	0.873	(0.543 -- 1.404)	0.577	827.232	79	266
SuperLearner (SL)	0.931	(0.598 -- 1.450)	0.753	1179.490	79	266
GBM	0.834	(0.529 -- 1.316)	0.436	1380.892	79	266

Subgroups - PCT ≥ 2 ng/ml (n=68)

Black font: Adjustment without centre as strata due to sample size.

Cox model	HR	CI	p-value	AIC	# events	# observations
Crude	0.733	(0.358 --1.504)	0.397	237.093	30	68
p-value based selection using values at baseline, the first ten days and differences	2.005	(0.743 – 5.408)	0.170	217.214	29	67
Liberal	1.035	(0.463 -- 2.312)	0.933	222.564	29	67
Restrictive	0.997	(0.447 -- 2.225)	0.994	239.933	30	68
SuperLearner (SL)	0.732	(0.361 -- 1.485)	0.388	299.747	30	68
GBM	0.674	(0.329 -- 1.383)	0.282	337.706	30	68

Subgroups - With malignant comorbidity (n=64)

Black font: Adjustment without centre as strata due to sample size.

Cox model	HR	CI	p-value	AIC	# events	# observations
Crude	0.758	(0.352 -- 1.632)	0.479	225.600	29	64
p-value based selection using values at baseline, the first ten days and differences	0.310	(0.056 -- 1.715)	0.180	133.351	25	60
Liberal	0.992	(0.375 – 2.623)	0.986	163.632	24	55
Restrictive	0.763	(0.340 -- 1.709)	0.510	221.997	29	64
SuperLearner (SL)	0.854	(0.362 -- 1.569)	0.650	494.308	29	64
GBM	0.769	(0.362 -- 1.632)	0.494	324.220	29	64

Subgroups - Without malignant comorbidity (n=252)

Black font: Adjustment without centre as strata due to sample size.

Cox model	HR	CI	p-value	AIC	# events	# observations
Crude	0.680	(0.411 -- 1.127)	0.135	706.784	67	252
p-value based selection using values at baseline, the first ten days and differences	0.608	(0.359 -- 1.030)	0.064	653.096	67	249
Liberal	0.557	(0.324 -- 0.957)	0.034	652.142	65	250
Restrictive	0.657	(0.392 -- 1.102)	0.112	683.154	67	252
SuperLearner (SL)	0.681	(0.410 -- 1.129)	0.136	871.833	67	252
GBM	0.602	(0.357 -- 1.017)	0.058	1156.813	67	252
