# Supplementary material for: Intravenous IgM-enriched immunoglobulins in critical COVID-19: a multicentre propensity-weighted cohort study
Source: Crit Care. 2022 Jul 7;26:204. doi: 10.1186/s13054-022-04059-0 (PMC9260992; doi:10.1186/s13054-022-04059-0)
Supplement: Supplementary file 2 — Additional file 2 Covariates included in the models. [file 13054_2022_4059_MOESM2_ESM.pdf]

<u>Additional file 2:</u>

Covariates included in the models

Baseline characteristics
- Participating study centre<br>- Age<br>- Sex<br>- Body mass index (BMI)<br>- Admission status (in-hospital vs. secondary transfer or emergency/direct admission)
**Underlying diseases**
- Arterial hypertension<br>- Cardiovascular diseases<br>- Heart failure<br>- Chronic obstructive pulmonary disease (COPD)<br>- Other pulmonary diseases<br>- Nicotine abuse<br>- Organ transplantation<br>- Alcoholism<br>- Chronical renal failure + dialysis requirement in case of terminally renal insufficiency<br>- Diabetes mellitus + insulin dependence<br>- Malignant underlying disease
**Long-term medications**
- ACE inhibitor<br>- AT2 receptor blockers<br>- Beta blockers<br>- Platelet inhibitors<br>- Marcumar, new oral anticoagulants (NOACs)<br>- Corticosteroids<br>- Immunosuppressants<br>- Opioids<br>- Indicator for and number of other medications<br>- Polypharmacy indicator ($\geq$ 5 medications)

**Number of days between symptom onset and ICU admission**
**Relevant laboratory parameters**<br>*(values at ICU admission, on the worst day in the first 10 days and the difference value between those time points)*
- Leukocytes, absolute<br>- Procalcitonin (PCT)<br>- D-dimer<br>- Hemoglobin
**Disease severity**
- Respiratory status (spontaneous/mask, nasal high flow, non-invasive ventilation (NIV), intubated)<br>*(status on ICU admission and on the worst day within the first 10 days in the ICU)*<br>- Highest level of respiratory care during ICU stay (non-invasive ventilation (NIV), controlled ventilation, extracorporeal membrane oxygenation (ECMO))<br>- Scores:<br>– Murray-Lung-Injury-Score (MLI)<br>*(score at ICU admission, on the worst day in the first 10 days in the ICU and the difference of the score between those time points)*<br>– Sequential Organ Failure Assessment (SOFA) Score<br>*(score at ICU admission, on the worst day in the first 10 days in the ICU and the difference of the score between those time points)*<br>– Acute Physiology And Chronic Health Evaluation (APACHE)-II-Score<br>*(score at ICU admission)*<br>– Acute kidney injury (AKI stage according to KDIGO)<br>*(at ICU admission and on the worst day in the first 10 days in the ICU)*<br>– Dialysis status<br>*(at ICU admission and on the worst day in the first 10 days in the ICU)*
**Adjuvant COVID-19 therapies**
– Glucocorticoids<br>– IL6 receptor antagonists<br>– Remdesivir<br>– Convalescent plasma/specific antibodies<br>– Others
