# Supplementary material for: Intravenous IgM-enriched immunoglobulins in critical COVID-19: a multicentre propensity-weighted cohort study
Source: Crit Care. 2022 Jul 7;26:204. doi: 10.1186/s13054-022-04059-0 (PMC9260992; doi:10.1186/s13054-022-04059-0)
Supplement: Supplementary file 3 — Additional file 3 Baseline characteristics of investigated subgroups. [file 13054_2022_4059_MOESM3_ESM.pdf]

Additional file 3

Table S1: Baseline characteristics for subgroup Ferritin ≥ 400µg/l, CRP ≥ 70mg/l, IL-6 ≥ 100pg/ml (n=131).

	Control group (N=63)	IGAM group (N=68)	p value
**Demographics**			
Age [years], mean (±SD)	60.5 (11.4)	56.8 (12.5)	0.076
Female sex, n (%)	11 (17.5%)	18 (26.5%)	0.303
Body mass index [kg/m^2], mean (±SD)	31.8 (7.47)	30.4 (5.91)	0.229
**Comorbidities, n (%)**			
None	12 (19.0%)	16 (23.5%)	0.680
Hypertension	42 (66.7%)	35 (51.5%)	0.112
Cardiovascular disease	12 (19.0%)	11 (16.2%)	0.840
Chronic heart failure	7 (11.1%)	4 (5.88%)	0.446
Chronic kidney disease	7 (11.1%)	4 (5.88%)	0.446
Chronic obstructive pulmonary disease	5 (7.94%)	6 (8.82%)	1.000
Diabetes mellitus	17 (27.0%)	13 (19.1%)	0.388
Malignant disease	4 (6.35%)	15 (22.1%)	0.021
**Permanent medication, n (%)**			
None	25 (39.7%)	22 (32.4%)	0.489
ACEI	12 (19.0%)	7 (10.3%)	0.241
ARB´s	12 (19.0%)	12 (17.6%)	1.000
Beta blockers	15 (23.8%)	19 (27.9%)	0.734
Platelet aggregation inhibitors	9 (14.3%)	8 (11.8%)	0.866
Anticoagulants	5 (7.94%)	5 (7.35%)	1.000
Corticosteroids	8 (12.7%)	9 (13.2%)	1.000
Immunosuppressive agents	4 (6.35%)	8 (11.8%)	0.441
Polypharmacy (≥ 5 drugs)	18 (28.6%)	14 (20.6%)	0.390
**COVID-19 course** - day with highest disease severity*			
Days after ICU admission [days], median (IQR)	3.90 (3.65)	4.96 (3.56)	0.098
Respiratory Support			0.262
- Supplemental oxygen, n(%)	2 (3.17%)	0 (0.00%)	
- High-flow oxygen device, n(%)	3 (4.76%)	1 (1.47%)	
- Non-invasive ventilation, n(%)	6 (9.52%)	4 (5.88%)	
- Mechanical ventilation, n(%)	52 (82.5%)	63 (92.6%)	
Horowitz index [PaO2/FiO2], median (IQR)	130 (83.7)	97.1 (46.6)	0.007
P_{insp} [cmH_2O], median (IQR)	27.2 (5.60)	28.0 (7.11)	0.537
PEEP [cmH_2O], median (IQR)	12.2 (3.39)	12.5 (3.23)	0.602
Murray Score; median (IQR)	11.6 (3.03)	12.9 (2.39)	0.015
SOFA Score; median (IQR)	10.3 (4.09)	11.3 (3.93)	0.174
AKI KDIGO stage, n (%)			0.952

-No acute renal injury	31 (49.2%)	34 (50.0%)	
-1	10 (15.9%)	10 (14.7%)	
-2	3 (4.76%)	5 (7.35%)	
-3	19 (30.2%)	19 (27.9%)	
Vasopressor support, n (%)	51 (81.0%)	59 (86.8%)	0.504
SARS-CoV-2 virus load [CT value]; mean (±SD)	31.1 (6.71)	25.5 (7.95)	0.033
Laboratory values - of day with highest disease severity*; median (IQR)			
Leukocyte count [1000/µL]	12.3 [9.55;16.6]	11.2 [8.30;21.4]	0.856
- Neutrophile count [1000/µL]	9.94 [7.10;14.1]	8.72 [6.35;15.3]	0.696
- Lymphocyte count [1000/µL]	0.90 [0.60;1.40]	0.98 [0.58;1.54]	0.625
C reactive protein [mg/L]	177 [113;267]	184 [130;284]	0.301
Procalcitonin [ng/mL]	0.68 [0.39;1.71]	1.08 [0.45;2.28]	0.197
Interleukin-6 [pg/mL]	151 [76.0;603]	275 [93.7;878]	0.084
Ferritin [µg/L]	1370 [911;2510]	1860 [1130;4397]	0.073
Platelet count [1000/µL]	245 [144;338]	179 [110;319]	0.075
Serum creatinine [mg/dL]	1.00 [0.75;1.71]	1.14 [0.74;1.65]	0.838
D-dimers [µg/mL]	2.22 [0.60;6.50]	2.87 [1.77;8.90]	0.081
Total bilirubin [mg/dL]	0.60 [0.37;1.30]	1.04 [0.60;2.27]	0.015
IgM serum concentration [mg/dL]	72.5 [61.5;140]	95.0 [94.0;126]	0.273
IgA serum concentration [mg/dL]	205 [164;292]	343 [215;482]	0.167
IgG serum concentration [mg/dL]	970 [748;986]	1180 [1080;1300]	0.088
**Adjunctive therapies**; n (%)			
Corticosteroids	50 (79.4%)	59 (86.8%)	0.369
Interleukin-6 receptor antagonist	14 (22.2%)	9 (13.2%)	0.262
Remdesivir	16 (25.4%)	11 (16.2%)	0.277

ACEI: Angiotensin converting-enzyme inhibitor; ARB: Angiotensin II receptor blocker; ICU: Intensive care unit; CT: cycle threshold; PaO_2: Partial pressure of oxygen; FiO_2: Fraction of inspired oxygen; PEEP:Positive end expiratory pressure; P_{insp}: Inspiratory plateau pressure; SOFA-Score: Sequential Organ Failure Assessment Score ; AKI: Acute kidney injury; KDIGO: Kidney Disease: Improving Global Outcomes; * Day of the most critical medical condition within the first 10 days after ICU admission.

Table S2: Baseline characteristics for subgroup Ferritin ≥ 1000µg/l, CRP ≥ 100mg/l, IL-6 ≥ 600pg/ml (n=98).

	**Control group (N=63)**	**IGAM group (N=68)**	**p value**
**Demographics**			
Age [years], mean (±SD)	62.0 (11.6)	56.1 (12.9)	0.019
Female sex, n (%)	7 (16.7%)	14 (25.0%)	0.456
Body mass index [kg/m^2], mean (±SD)	31.4 (6.95)	30.4 (5.67)	0.460
**Comorbidities, n (%)**			
None	8 (19.0%)	14 (25.0%)	0.650
Hypertension	28 (66.7%)	28 (50.0%)	0.149

Cardiovascular disease	9 (21.4%)	8 (14.3%)	0.513
Chronic heart failure	3 (7.14%)	3 (5.36%)	1.000
Chronic kidney disease	6 (14.3%)	4 (7.14%)	0.318
Chronic obstructive pulmonary disease	3 (7.14%)	4 (7.14%)	1.000
Diabetes mellitus	11 (26.2%)	9 (16.1%)	0.329
Malignant disease	3 (7.14%)	14 (25.0%)	0.041
**Permanent medication, n (%)**			
None	20 (47.6%)	21 (37.5%)	0.425
ACEI	8 (19.0%)	5 (8.93%)	0.246
ARB´s	8 (19.0%)	12 (21.4%)	0.971
Beta blockers	9 (21.4%)	17 (30.4%)	0.448
Platelet aggregation inhibitors	3 (7.14%)	6 (10.7%)	0.728
Anticoagulants	2 (4.76%)	2 (3.57%)	1.000
Corticosteroids	5 (11.9%)	7 (12.5%)	1.000
Immunosuppressive agents	1 (2.38%)	8 (14.3%)	0.074
Polypharmacy (≥ 5 drugs)	10 (23.8%)	11 (19.6%)	0.804
**COVID-19 course** - day with highest disease severity*			
Days after ICU admission [days], median (IQR)	3.71 (3.66)	5.18 (3.50)	0.049
Respiratory Support			0.405
- Supplemental oxygen, n(%)	2 (4.76%)	0 (0.00%)	
- High-flow oxygen device, n(%)	2 (4.76%)	1 (1.79%)	
- Non-invasive ventilation, n(%)	2 (4.76%)	3 (5.36%)	
- Mechanical ventilation, n(%)	36 (85.7%)	52 (92.9%)	
Horowitz index [PaO2/FiO2], median (IQR)	126 (84.7)	97.4 (48.2)	0.051
P_{insp} [cmH_2O], median (IQR)	27.1 (5.36)	28.2 (7.35)	0.430
PEEP [cmH_2O], median (IQR)	12.5 (2.93)	12.4 (3.47)	0.850
Murray Score; median (IQR)	11.8 (3.07)	12.8 (2.59)	0.118
SOFA Score; median (IQR)	11.1 (4.05)	11.6 (3.96)	0.519
AKI KDIGO stage, n (%)			0.581
-No acute renal injury	20 (47.6%)	24 (42.9%)	
-1	3 (7.14%)	9 (16.1%)	
-2	3 (7.14%)	5 (8.93%)	
-3	16 (38.1%)	18 (32.1%)	
Vasopressor support, n (%)	36 (85.7%)	48 (85.7%)	1.000
SARS-CoV-2 virus load [CT value]; mean (±SD)	33.1 (6.20)	25.2 (8.57)	0.014
Laboratory values - of day with highest disease severity*; median (IQR)			
Leukocyte count [1000/µL]	12.3 [8.60;18.7]	12.7 [8.63;22.6]	0.804
- Neutrophile count [1000/µL]	10.0 [6.72;14.7]	9.54 [6.61;15.7]	0.995
- Lymphocyte count [1000/µL]	0.91 [0.67;1.40]	0.98 [0.56;1.50]	0.848
C reactive protein [mg/L]	194 [137;290]	186 [132;284]	0.969
Procalcitonin [ng/mL]	0.72 [0.44;1.71]	1.31 [0.36;3.20]	0.358
Interleukin-6 [pg/mL]	262 [90.3;800]	333 [90.2;1290]	0.722

Ferritin [µg/L]	1845 [1296;2995]	2317 [1484;5779]	0.144
Platelet count [1000/µL]	183 [133;326]	209 [111;319]	0.494
Serum creatinine [mg/dL]	1.18 [0.78;1.80]	1.20 [0.80;1.79]	0.833
D-dimers [µg/mL]	2.36 [1.10;7.23]	3.61 [2.01;9.62]	0.111
Total bilirubin [mg/dL]	0.74 [0.42;1.30]	1.15 [0.60;2.40]	0.015
IgM serum concentration [mg/dL]	82.0 [61.0;160]	126 [102;196]	0.456
IgA serum concentration [mg/dL]	222 [152;310]	343 [279;442]	0.197
IgG serum concentration [mg/dL]	972 [787;993]	1080 [886;1280]	0.439
**Adjunctive therapies**; n (%)			
Corticosteroids	35 (83.3%)	49 (87.5%)	0.771
Interleukin-6 receptor antagonist	8 (19.0%)	7 (12.5%)	0.544
Remdesivir	10 (23.8%)	8 (14.3%)	0.347

ACEI: Angiotensin converting-enzyme inhibitor; ARB: Angiotensin II receptor blocker; ICU: Intensive care unit; CT: cycle threshold; PaO_2: Partial pressure of oxygen; FiO_2: Fraction of inspired oxygen; PEEP:Positive end expiratory pressure; P_{insp}: Inspiratory plateau pressure; SOFA-Score: Sequential Organ Failure Assessment Score ; AKI: Acute kidney injury; KDIGO: Kidney Disease: Improving Global Outcomes; * Day of the most critical medical condition within the first 10 days after ICU admission.

Table S3: Baseline characteristics for subgroup CRP ≥ 70mg/L or IgM ≤ 80mg/dL (n=266).

	Control group (N=142)	IGAM group (N=124)	p value
**Demographics**			
Age [years], mean (±SD)	61.9 (11.9)	58.7 (12.1)	0.031
Female sex, n (%)	39 (27.5%)	33 (26.6%)	0.986
Body mass index [kg/m^2], mean (±SD)	32.2 (7.33)	30.2 (5.87)	0.019
**Comorbidities, n (%)**			
None	20 (14.1%)	24 (19.4%)	0.323
Hypertension	101 (71.1%)	71 (57.3%)	0.026
Cardiovascular disease	42 (29.6%)	26 (21.0%)	0.143
Chronic heart failure	11 (7.75%)	13 (10.5%)	0.574
Chronic kidney disease	14 (9.86%)	14 (11.3%)	0.858
Chronic obstructive pulmonary disease	9 (6.34%)	10 (8.06%)	0.759
Diabetes mellitus	51 (35.9%)	32 (25.8%)	0.100
Malignant disease	10 (7.04%)	28 (22.6%)	0.001
**Permanent medication, n (%)**			
None	42 (29.6%)	37 (29.8%)	1.000
ACEI	38 (26.8%)	26 (21.0%)	0.338
ARB´s	33 (23.2%)	15 (12.1%)	0.028
Beta blockers	45 (31.7%)	41 (33.1%)	0.914
Platelet aggregation inhibitors	35 (24.6%)	21 (16.9%)	0.165
Anticoagulants	14 (9.86%)	9 (7.26%)	0.593
Corticosteroids	17 (12.0%)	14 (11.3%)	1.000
Immunosuppressive agents	6 (4.23%)	15 (12.1%)	0.032

Polypharmacy (≥ 5 drugs)	59 (41.5%)	41 (33.1%)	0.194
**COVID-19 course** - day with highest disease severity*			
Days after ICU admission [days], median (IQR)	3.85 (3.58)	4.81 (3.46)	0.027
Respiratory Support			0.644
- Supplemental oxygen, n(%)	3 (2.11%)	2 (1.61%)	
- High-flow oxygen device, n(%)	11 (7.75%)	5 (4.03%)	
- Non-invasive ventilation, n(%)	8 (5.63%)	6 (4.84%)	
- Mechanical ventilation, n(%)	120 (84.5%)	111 (89.5%)	
Horowitz index [PaO2/FiO2], median (IQR)	126 (66.7)	107 (67.2)	0.027
P_{insp} [cmH_2O], median (IQR)	26.4 (5.96)	27.2 (6.14)	0.282
PEEP [cmH_2O], median (IQR)	11.7 (3.43)	12.2 (2.94)	0.199
Murray Score; median (IQR)	11.3 (3.09)	12.2 (2.90)	0.014
SOFA Score; median (IQR)	9.42 (3.86)	10.8 (3.86)	0.005
AKI KDIGO stage, n (%)			0.195
-No acute renal injury	86 (60.6%)	64 (51.6%)	
-1	18 (12.7%)	12 (9.68%)	
-2	4 (2.82%)	7 (5.65%)	
-3	34 (23.9%)	41 (33.1%)	
Vasopressor support, n (%)	107 (75.4%)	106 (85.5%)	0.056
SARS-CoV-2 virus load [CT value]; mean (±SD)	30.3 (6.49)	24.5 (7.37)	<0.001
Laboratory values - of day with highest disease severity*; median (IQR)			
Leukocyte count [1000/µL]	11.7 [7.92;16.1]	12.1 [8.54;20.3]	0.312
- Neutrophile count [1000/µL]	9.50 [6.50;13.3]	9.54 [6.50;15.7]	0.685
- Lymphocyte count [1000/µL]	0.90 [0.56;1.40]	0.98 [0.52;1.48]	0.921
C reactive protein [mg/L]	166 [116;235]	186 [130;271]	0.029
Procalcitonin [ng/mL]	0.57 [0.25;1.32]	1.15 [0.47;3.38]	<0.001
Interleukin-6 [pg/mL]	136 [59.6;368]	247 [91.1;701]	0.011
Ferritin [µg/L]	1268 [728;1966]	2037 [1129;4577]	<0.001
Platelet count [1000/µL]	250 [160;336]	179 [108;284]	0.001
Serum creatinine [mg/dL]	0.93 [0.70;1.67]	1.10 [0.70;1.69]	0.257
D-dimers [µg/mL]	2.46 [1.27;5.53]	2.90 [1.50;8.57]	0.197
Total bilirubin [mg/dL]	0.60 [0.40;1.01]	0.84 [0.60;1.90]	0.001
IgM serum concentration [mg/dL]	75.0 [59.0;100]	94.5 [77.2;130]	0.170
IgA serum concentration [mg/dL]	234 [177;295]	210 [190;447]	0.489
IgG serum concentration [mg/dL]	869 [758;971]	1070 [699;1270]	0.262
**Adjunctive therapies**; n (%)			
Corticosteroids	112 (78.9%)	106 (85.5%)	0.215
Interleukin-6 receptor antagonist	18 (12.7%)	7 (5.65%)	0.080
Remdesivir	29 (20.4%)	13 (10.5%)	0.040

ACEI: Angiotensin converting-enzyme inhibitor; ARB: Angiotensin II receptor blocker; ICU: Intensive care unit; CT: cycle threshold; PaO_2: Partial pressure of oxygen; FiO_2: Fraction of inspired oxygen; PEEP:Positive end expiratory pressure; P_{insp}: Inspiratory plateau pressure; SOFA-Score: Sequential Organ Failure Assessment Score ; AKI: Acute kidney injury; KDIGO: Kidney Disease: Improving Global Outcomes; * Day of the most critical medical condition within the first 10 days after ICU admission.

<u>Table S4:</u> Baseline characteristics for subgroup PCT ≥ 2 ng/ml (n=68).

	Control group (N=28)	IGAM group (N=40)	p value
**Demographics**			
Age [years], mean (±SD)	59.3 (13.9)	57.9 (11.9)	0.650
Female sex, n (%)	8 (28.6%)	8 (20.0%)	0.569
Body mass index [kg/m^2], mean (±SD)	34.3 (7.28)	30.2 (5.70)	0.016
**Comorbidities, n (%)**			
None	7 (25.0%)	5 (12.5%)	0.211
Hypertension	17 (60.7%)	24 (60.0%)	1.000
Cardiovascular disease	5 (17.9%)	9 (22.5%)	0.872
Chronic heart failure	4 (14.3%)	8 (20.0%)	0.748
Chronic kidney disease	6 (21.4%)	12 (30.0%)	0.611
Chronic obstructive pulmonary disease	1 (3.57%)	2 (5.00%)	1.000
Diabetes mellitus	7 (25.0%)	11 (27.5%)	1.000
Malignant disease	3 (10.7%)	9 (22.5%)	0.334
**Permanent medication, n (%)**			
None	13 (46.4%)	11 (27.5%)	0.177
ACEI	3 (10.7%)	10 (25.0%)	0.246
ARB´s	5 (17.9%)	7 (17.5%)	1.000
Beta blockers	8 (28.6%)	15 (37.5%)	0.613
Platelet aggregation inhibitors	4 (14.3%)	8 (20.0%)	0.748
Anticoagulants	3 (10.7%)	2 (5.00%)	0.396
Corticosteroids	1 (3.57%)	9 (22.5%)	0.039
Immunosuppressive agents	1 (3.57%)	10 (25.0%)	0.021
Polypharmacy (≥ 5 drugs)	8 (28.6%)	21 (52.5%)	0.086
**COVID-19 course** - day with highest disease severity*			
Days after ICU admission [days], median (IQR)	4.57 (3.32)	5.25 (3.66)	0.429
Respiratory Support			0.419
- Supplemental oxygen, n(%)	0 (0.00%)	2 (5.00%)	
- High-flow oxygen device, n(%)	0 (0.00%)	2 (5.00%)	
- Non-invasive ventilation, n(%)	0 (0.00%)	1 (2.50%)	
- Mechanical ventilation, n(%)	28 (100%)	35 (87.5%)	
Horowitz index [PaO2/FiO2], median (IQR)	120 (59.0)	119 (91.1)	0.981
P_{insp} [cmH_2O], median (IQR)	27.5 (4.97)	26.6 (6.90)	0.582
PEEP [cmH_2O], median (IQR)	13.2 (3.84)	12.4 (3.01)	0.333
Murray Score; median (IQR)	12.0 (2.36)	12.0 (3.62)	0.931
SOFA Score; median (IQR)	13.8 (3.24)	12.1 (3.78)	0.055
AKI KDIGO stage, n (%)			0.444
-No acute renal injury	3 (10.7%)	9 (22.5%)	
-1	4 (14.3%)	4 (10.0%)	
-2	1 (3.57%)	4 (10.0%)	

-3	20 (71.4%)	23 (57.5%)	
Vasopressor support, n (%)	26 (92.9%)	34 (85.0%)	0.455
SARS-CoV-2 virus load [CT value]; mean (±SD)	31.8 (6.49)	22.5 (5.89)	<0.001
Laboratory values - of day with highest disease severity*; median (IQR)			
Leukocyte count [1000/µL]	16.9 [14.2;26.0]	11.4 [6.95;18.6]	0.003
- Neutrophile count [1000/µL]	17.1 [10.9;23.9]	9.74 [5.82;16.6]	0.037
- Lymphocyte count [1000/µL]	0.80 [0.47;1.90]	0.82 [0.40;1.27]	0.500
C reactive protein [mg/L]	200 [123;322]	210 [130;297]	0.975
Procalcitonin [ng/mL]	6.32 [3.39;15.1]	7.34 [3.45;15.5]	0.803
Interleukin-6 [pg/mL]	416 [169;816]	509 [290;1500]	0.582
Ferritin [µg/L]	1788 [1034;3218]	2476 [1081;7578]	0.321
Platelet count [1000/µL]	233 [93.5;280]	141 [78.5;221]	0.051
Serum creatinine [mg/dL]	1.89 [1.32;2.80]	1.90 [1.34;3.22]	0.940
D-dimers [µg/mL]	3.38 [1.48;6.55]	5.66 [2.05;9.93]	0.289
Total bilirubin [mg/dL]	1.30 [0.66;3.40]	0.91 [0.60;2.00]	0.330
IgM serum concentration [mg/dL]	57.0 [57.0;57.0]	172 [125;218]	0.221
IgA serum concentration [mg/dL]	189 [189;189]	364 [277;452]	0.480
IgG serum concentration [mg/dL]	999 [846;1035]	1016 [785;1248]	1.000
**Adjunctive therapies**; n (%)			
Corticosteroids	22 (78.6%)	33 (82.5%)	0.927
Interleukin-6 receptor antagonist	6 (21.4%)	3 (7.50%)	0.146
Remdesivir	5 (17.9%)	3 (7.50%)	0.259

ACEI: Angiotensin converting-enzyme inhibitor; ARB: Angiotensin II receptor blocker; ICU: Intensive care unit; CT: cycle threshold; PaO_2: Partial pressure of oxygen; FiO_2: Fraction of inspired oxygen; PEEP:Positive end expiratory pressure; P_{insp}: Inspiratory plateau pressure; SOFA-Score: Sequential Organ Failure Assessment Score ; AKI: Acute kidney injury; KDIGO: Kidney Disease: Improving Global Outcomes; * Day of the most critical medical condition within the first 10 days after ICU admission.

Table S5: Baseline characteristics for subgroup spontaneous breathing (n=68).

	Control group (N=28)	IGAM group (N=22)	p value
**Demographics**			
Age [years], mean (±SD)	67.3 (9.53)	62.1 (13.9)	0.146
Female sex, n (%)	3 (10.7%)	2 (9.09%)	1.000
Body mass index [kg/m^2], mean (±SD)	29.4 (4.93)	29.4 (6.11)	0.967
**Comorbidities, n (%)**			
None	1 (3.57%)	4 (18.2%)	0.155
Hypertension	22 (78.6%)	13 (59.1%)	0.238
Cardiovascular disease	12 (42.9%)	10 (45.5%)	1.000
Chronic heart failure	4 (14.3%)	5 (22.7%)	0.481
Chronic kidney disease	6 (21.4%)	5 (22.7%)	1.000
Chronic obstructive pulmonary disease	4 (14.3%)	2 (9.09%)	0.683
Diabetes mellitus	11 (39.3%)	6 (27.3%)	0.556

Malignant disease	5 (17.9%)	5 (22.7%)	0.732
**Permanent medication, n (%)**			
None	3 (10.7%)	5 (22.7%)	0.277
ACEI	10 (35.7%)	4 (18.2%)	0.292
ARB´s	7 (25.0%)	1 (4.55%)	0.064
Beta blockers	7 (25.0%)	9 (40.9%)	0.373
Platelet aggregation inhibitors	9 (32.1%)	7 (31.8%)	1.000
Anticoagulants	6 (21.4%)	4 (18.2%)	1.000
Corticosteroids	3 (10.7%)	5 (22.7%)	0.277
Immunosuppressive agents	1 (3.57%)	2 (9.09%)	0.576
Polypharmacy (≥ 5 drugs)	13 (46.4%)	9 (40.9%)	0.918
**COVID-19 course** - day with highest disease severity*			
Days after ICU admission [days], median (IQR)	1.57 (2.35)	3.59 (3.22)	0.018
Respiratory Support			0.160
- Supplemental oxygen, n(%)	3 (10.7%)	3 (13.6%)	
- High-flow oxygen device, n(%)	15 (53.6%)	6 (27.3%)	
- Non-invasive ventilation, n(%)	10 (35.7%)	13 (59.1%)	
- Mechanical ventilation, n(%)	n/a	n/a	
Horowitz index [PaO2/FiO2], median (IQR)	132 (73.8)	155 (128)	0.469
P_{insp} [cmH_2O], median (IQR)	n/a	n/a	.
PEEP [cmH_2O], median (IQR)	7.20 (2.04)	7.73 (1.45)	0.496
Murray Score; median (IQR)	7.06 (1.39)	6.92 (2.78)	0.876
SOFA Score; median (IQR)	6.00 (2.72)	7.05 (3.09)	0.218
AKI KDIGO stage, n (%)			0.895
-No acute renal injury	14 (50.0%)	13 (59.1%)	
-1	7 (25.0%)	4 (18.2%)	
-2	1 (3.57%)	1 (4.55%)	
-3	6 (21.4%)	4 (18.2%)	
Vasopressor support, n (%)	5 (17.9%)	7 (31.8%)	0.416
SARS-CoV-2 virus load [CT value]; mean (±SD)	27.2 (8.54)	24.8 (1.75)	0.604
Laboratory values - of day with highest disease severity*; median (IQR)			
Leukocyte count [1000/µL]	9.93 [6.50;12.1]	9.90 [5.80;14.4]	0.860
- Neutrophile count [1000/µL]	8.07 [5.35;9.76]	7.64 [4.51;9.85]	0.647
- Lymphocyte count [1000/µL]	0.53 [0.37;0.91]	1.00 [0.45;1.20]	0.154
C reactive protein [mg/L]	116 [74.3;184]	106 [37.6;139]	0.099
Procalcitonin [ng/mL]	0.20 [0.11;0.92]	0.32 [0.16;1.32]	0.306
Interleukin-6 [pg/mL]	151 [78.9;344]	91.4 [40.4;290]	0.343
Ferritin [µg/L]	1291 [611;1736]	898 [373;1418]	0.361
Platelet count [1000/µL]	260 [198;339]	244 [148;312]	0.328
Serum creatinine [mg/dL]	0.78 [0.67;1.88]	1.00 [0.73;1.33]	0.755
D-dimers [µg/mL]	1.44 [0.50;2.26]	1.62 [0.63;3.00]	0.468
Total bilirubin [mg/dL]	0.52 [0.40;0.98]	0.61 [0.38;0.84]	0.867

IgM serum concentration [mg/dL]	64.0 [51.8;72.5]	72.0 [66.0;77.2]	0.242
IgA serum concentration [mg/dL]	197 [140;224]	198 [186;240]	0.593
IgG serum concentration [mg/dL]	776 [737;916]	702 [654;808]	0.394
**Adjunctive therapies**; n (%)			
Corticosteroids	26 (92.9%)	16 (72.7%)	0.116
Interleukin-6 receptor antagonist	5 (17.9%)	3 (13.6%)	1.000
Remdesivir	9 (32.1%)	10 (45.5%)	0.503

ACEI: Angiotensin converting-enzyme inhibitor; ARB: Angiotensin II receptor blocker; ICU: Intensive care unit; CT: cycle threshold; PaO_2: Partial pressure of oxygen; FiO_2: Fraction of inspired oxygen; PEEP:Positive end expiratory pressure; P_{insp}: Inspiratory plateau pressure; SOFA-Score: Sequential Organ Failure Assessment Score ; AKI: Acute kidney injury; KDIGO: Kidney Disease: Improving Global Outcomes; * Day of the most critical medical condition within the first 10 days after ICU admission.

Table S6: Baseline characteristics for subgroup IGAM dosage >15g/d for at least 3 days (n=283).

	Control group (N=170)	IGAM group (N=113)	p value
**Demographics**			
Age [years], mean (±SD)	62.5 (11.6)	58.9 (12.0)	0.045
Female sex, n (%)	43 (25.3%)	30 (26.5%)	0.922
Body mass index [kg/m^2], mean (±SD)	31.9 (7.18)	30.1 (5.54)	0.063
**Comorbidities, n (%)**			
None	26 (15.3%)	20 (17.7%)	1.000
Hypertension	115 (67.6%)	65 (57.5%)	0.324
Cardiovascular disease	48 (28.2%)	27 (23.9%)	0.908
Chronic heart failure	13 (7.65%)	13 (11.5%)	0.557
Chronic kidney disease	17 (10.0%)	15 (13.3%)	0.509
Chronic obstructive pulmonary disease	10 (5.88%)	12 (10.6%)	0.448
Diabetes mellitus	60 (35.3%)	35 (31.0%)	0.532
Malignant disease	14 (8.24%)	24 (21.2%)	0.005
**Permanent medication, n (%)**			
None	51 (30.0%)	31 (27.4%)	1.000
ACEI	46 (27.1%)	21 (18.6%)	0.342
ARB´s	33 (19.4%)	16 (14.2%)	0.365
Beta blockers	54 (31.8%)	39 (34.5%)	0.758
Platelet aggregation inhibitors	43 (25.3%)	26 (23.0%)	1.000
Anticoagulants	18 (10.6%)	9 (7.96%)	0.763
Corticosteroids	18 (10.6%)	17 (15.0%)	0.785
Immunosuppressive agents	7 (4.12%)	14 (12.4%)	0.054
Polypharmacy (≥ 5 drugs)	71 (41.8%)	41 (36.3%)	0.355
**COVID-19 course** - day with highest disease severity*			
Days after ICU admission [days], median (IQR)	3.00 [1.00;6.00]	5.00 [2.00;8.00]	0.031

Respiratory Support			0.338
- Supplemental oxygen, n(%)	3 (1.76%)	3 (2.65%)	
- High-flow oxygen device, n(%)	15 (8.82%)	4 (3.54%)	
- Non-invasive ventilation, n(%)	10 (5.88%)	6 (5.31%)	
- Mechanical ventilation, n(%)	142 (83.5%)	100 (88.5%)	
Horowitz index [PaO2/FiO2], median (IQR)	105 [75.2;163]	96.0 [71.0;147]	0.115
P_{insp} [cmH_2O], median (IQR)	27.0 [23.2;30.0]	27.0 [23.5;31.0]	1.000
PEEP [cmH_2O], median (IQR)	12.0 [10.0;14.0]	12.0 [11.0;14.0]	0.036
Murray Score; median (IQR)	12.0 [9.00;14.0]	13.0 [10.0;14.0]	0.106
SOFA Score; median (IQR)	9.52 (3.92)	10.5 (3.76)	0.116
AKI KDIGO stage, n (%)			0.580
-No acute renal injury	100 (58.8%)	58 (51.3%)	
-1	21 (12.4%)	11 (9.73%)	
-2	6 (3.53%)	6 (5.31%)	
-3	43 (25.3%)	38 (33.6%)	
Vasopressor support, n (%)	129 (75.9%)	92 (81.4%)	0.270
SARS-CoV-2 virus load [CT value]; mean (±SD)	30.1 (6.02)	25.5 (6.85)	0.003
Laboratory values - of day with highest disease severity*; median (IQR)			
Leukocyte count [1000/µL]	11.7 [8.41;16.2]	12.7 [8.19;20.1]	0.556
- Neutrophile count [1000/µL]	9.55 [6.53;13.8]	9.80 [6.38;14.5]	0.973
- Lymphocyte count [1000/µL]	0.87 [0.49;1.40]	0.90 [0.51;1.56]	0.641
C reactive protein [mg/L]	149 [83.2;206]	151 [116;260]	0.168
Procalcitonin [ng/mL]	0.51 [0.20;1.20]	0.81 [0.35;2.16]	0.009
Interleukin-6 [pg/mL]	139 [59.6;376]	247 [79.9;720]	0.122
Ferritin [µg/L]	1304 [668;2237]	1638 [875;3987]	0.041
Platelet count [1000/µL]	246 [158;336]	182 [115;287]	0.008
Serum creatinine [mg/dL]	0.93 [0.69;1.56]	1.07 [0.74;1.71]	0.454
D-dimers [µg/mL]	2.37 [1.20;4.62]	2.65 [1.31;7.90]	0.500
Total bilirubin [mg/dL]	0.60 [0.40;1.06]	0.76 [0.50;1.90]	0.046
IgM serum concentration [mg/dL]	80.0 [63.0;115]	77.0 [41.0;126]	0.405
IgA serum concentration [mg/dL]	248 [189;327]	214 [159;284]	0.305
IgG serum concentration [mg/dL]	916 [784;1063]	877 [659;1080]	0.450
**Adjunctive therapies**; n (%)			
Corticosteroids	139 (81.8%)	94 (83.2%)	0.882
Interleukin-6 receptor antagonist	22 (12.9%)	7 (6.19%)	0.215
Remdesivir	36 (21.2%)	14 (12.4%)	0.155

ACEI: Angiotensin converting-enzyme inhibitor; ARB: Angiotensin II receptor blocker; ICU: Intensive care unit; CT: cycle threshold; PaO_2: Partial pressure of oxygen; FiO_2: Fraction of inspired oxygen; PEEP:Positive end expiratory pressure; P_{insp}: Inspiratory plateau pressure; SOFA-Score: Sequential Organ Failure Assessment Score ; AKI: Acute kidney injury; KDIGO: Kidney Disease: Improving Global Outcomes; * Day of the most critical medical condition within the first 10 days after ICU admission.

Table S7: Baseline characteristics for subgroup IGAM dosage <15g/d and/or <3 days (n=203).

	Control group (N=170)	IGAM group (N=33)	p value
**Demographics**			
Age [years], mean (±SD)	62.5 (11.6)	61.0 (15.1)	0.799
Female sex, n (%)	43 (25.3%)	6 (18.2%)	0.772
Body mass index [kg/m^2], mean (±SD)	31.9 (7.18)	30.6 (6.52)	0.507
**Comorbidities, n (%)**			
None	26 (15.3%)	6 (18.2%)	1.000
Hypertension	115 (67.6%)	21 (63.6%)	0.806
Cardiovascular disease	48 (28.2%)	10 (30.3%)	0.976
Chronic heart failure	13 (7.65%)	5 (15.2%)	0.544
Chronic kidney disease	17 (10.0%)	7 (21.2%)	0.239
Chronic obstructive pulmonary disease	10 (5.88%)	1 (3.03%)	1.000
Diabetes mellitus	60 (35.3%)	6 (18.2%)	0.258
Malignant disease	14 (8.24%)	10 (30.3%)	0.004
**Permanent medication, n (%)**			
None	51 (30.0%)	10 (30.3%)	1.000
ACEI	46 (27.1%)	10 (30.3%)	0.866
ARB´s	33 (19.4%)	2 (6.06%)	0.325
Beta blockers	54 (31.8%)	13 (39.4%)	0.758
Platelet aggregation inhibitors	43 (25.3%)	7 (21.2%)	1.000
Anticoagulants	18 (10.6%)	4 (12.1%)	0.763
Corticosteroids	18 (10.6%)	4 (12.1%)	0.785
Immunosuppressive agents	7 (4.12%)	4 (12.1%)	0.124
Polypharmacy (≥ 5 drugs)	71 (41.8%)	12 (36.4%)	0.645
COVID-19 course - day with highest disease severity*			
Days after ICU admission [days], median (IQR)	3.00 [1.00;6.00]	3.00 [1.00;7.00]	0.310
Respiratory Support			0.076
- Supplemental oxygen, n(%)	3 (1.76%)	0 (0.00%)	
- High-flow oxygen device, n(%)	15 (8.82%)	2 (6.06%)	
- Non-invasive ventilation, n(%)	10 (5.88%)	7 (21.2%)	
- Mechanical ventilation, n(%)	142 (83.5%)	24 (72.7%)	
Horowitz index [PaO2/FiO2], median (IQR)	105 [75.2;163]	77.0 [69.0;94.0]	0.004
P_{insp} [cmH_2O], median (IQR)	27.0 [23.2;30.0]	28.0 [23.5;30.0]	1.000
PEEP [cmH2O], median (IQR)	12.0 [10.0;14.0]	10.0 [8.00;13.5]	0.006
Murray Score; median (IQR)	12.0 [9.00;14.0]	13.0 [10.5;13.0	0.755
SOFA Score; median (IQR)	9.52 (3.92)	10.4 (4.02)	0.996
AKI KDIGO stage, n (%)			0.580
-No acute renal injury	100 (58.8%)	15 (45.5%)	

-1	21 (12.4%)	6 (18.2%)	
-2	6 (3.53%)	2 (6.06%)	
-3	43 (25.3%)	10 (30.3%)	
Vasopressor support, n (%)	129 (75.9%)	21 (63.6%)	0.143
SARS-CoV-2 virus load [CT value]; mean (±SD)	30.1 (6.02)	21.0 (6.85)	<0.001
Laboratory values - of day with highest disease severity*; median (IQR)			
Leukocyte count [1000/µL]	11.7 [8.41;16.2]	10.6 [7.78;15.9]	0.556
- Neutrophile count [1000/µL]	9.55 [6.53;13.8]	7.81 [6.08;13.6]	0.455
- Lymphocyte count [1000/µL]	0.87 [0.49;1.40]	0.94 [0.37;1.14]	0.632
C reactive protein [mg/L]	149 [83.2;206]	170 [91.2;269]	0.701
Procalcitonin [ng/mL]	0.51 [0.20;1.20]	1.41 [0.28;7.48]	0.074
Interleukin-6 [pg/mL]	139 [59.6;376]	221 [94.4;825]	0.489
Ferritin [µg/L]	1304 [668;2237]	1718 [1120;4413]	0.054
Platelet count [1000/µL]	246 [158;336]	164 [128;230]	0.008
Serum creatinine [mg/dL]	0.93 [0.69;1.56]	1.20 [0.80;1.45]	0.454
D-dimers [µg/mL]	2.37 [1.20;4.62]	2.70 [1.90;5.66]	0.500
Total bilirubin [mg/dL]	0.60 [0.40;1.06]	0.84 [0.58;1.39]	0.188
IgM serum concentration [mg/dL]	80.0 [63.0;115]	51.0 [27.0;55.0]	0.006
IgA serum concentration [mg/dL]	248 [189;327]	174 [109;249]	0.144
IgG serum concentration [mg/dL]	916 [784;1063]	636 [354;858]	0.005
**Adjunctive therapies**; n (%)			
Corticosteroids	139 (81.8%)	31 (93.9%)	0.243
Interleukin-6 receptor antagonist	22 (12.9%)	5 (15.2%)	0.780
Remdesivir	36 (21.2%)	8 (24.2%)	0.873

ACEI: Angiotensin converting-enzyme inhibitor; ARB: Angiotensin II receptor blocker; ICU: Intensive care unit; CT: cycle threshold; PaO_2: Partial pressure of oxygen; FiO_2: Fraction of inspired oxygen; PEEP:Positive end expiratory pressure; P_{insp}: Inspiratory plateau pressure; SOFA-Score: Sequential Organ Failure Assessment Score ; AKI: Acute kidney injury; KDIGO: Kidney Disease: Improving Global Outcomes; * Day of the most critical medical condition within the first 10 days after ICU admission.

Table S8: Baseline characteristics for subgroup with malignancies (n=64).

	**Control group (N=19)**	**IGAM group (N=45)**	**p value**
**Demographics**			
Age [years], mean (±SD)	67.2 (9.15)	60.7 (13.9)	0.034
Female sex, n (%)	4 (21.1%)	15 (33.3%)	0.495
Body mass index [kg/m^2], mean (±SD)	29.6 (6.82)	29.4 (5.45)	0.939
**Comorbidities, n (%)**			
None	0 (0.00%)	2 (4.44%)	1.000
Hypertension	15 (78.9%)	28 (62.2%)	0.312
Cardiovascular disease	6 (31.6%)	14 (31.1%)	1.000

Chronic heart failure	2 (10.5%)	5 (11.1%)	1.000
Chronic kidney disease	4 (21.1%)	6 (13.3%)	0.466
Chronic obstructive pulmonary disease	2 (10.5%)	2 (4.44%)	0.576
Diabetes mellitus	6 (31.6%)	14 (31.1%)	1.000
Malignant disease	n/a	n/a	-
**Permanent medication, n (%)**			
None	1 (5.26%)	7 (15.6%)	0.418
ACEI	5 (26.3%)	10 (22.2%)	0.753
ARB´s	6 (31.6%)	7 (15.6%)	0.180
Beta blockers	7 (36.8%)	19 (42.2%)	0.903
Platelet aggregation inhibitors	7 (36.8%)	11 (24.4%)	0.482
Anticoagulants	6 (31.6%)	3 (6.67%)	0.016
Corticosteroids	7 (36.8%)	8 (17.8%)	0.117
Immunosuppressive agents	7 (36.8%)	18 (40.0%)	1.000
Polypharmacy (≥ 5 drugs)	12 (63.2%)	22 (48.9%)	0.441
COVID-19 course - day with highest disease severity*			
Days after ICU admission [days], median (IQR)	3.58 (3.67)	4.44 (3.17)	0.377
Respiratory Support			0.458
- Supplemental oxygen, n(%)	1 (5.26%)	2 (4.44%)	
- High-flow oxygen device, n(%)	3 (15.8%)	3 (6.67%)	
- Non-invasive ventilation, n(%)	1 (5.26%)	1 (2.22%)	
- Mechanical ventilation, n(%)	14 (73.7%)	39 (86.7%)	
Horowitz index [PaO2/FiO2], median (IQR)	123 (55.9)	130 (104)	0.721
P_{insp} [cmH_2O], median (IQR)	24.4 (8.22)	28.1 (7.39)	0.154
PEEP [cmH2O], median (IQR)	10.3 (2.71)	12.1 (3.19)	0.045
Murray Score; median (IQR)	10.3 (3.03)	11.3 (3.60)	0.312
SOFA Score; median (IQR)	9.95 (3.55)	10.2 (3.36)	0.776
AKI KDIGO stage, n (%)			0.698
-No acute renal injury	12 (63.2%)	22 (48.9%)	
-1	2 (10.5%)	9 (20.0%)	
-2	0 (0.00%)	1 (2.22%)	
-3	5 (26.3%)	13 (28.9%)	
Vasopressor support, n (%)	13 (68.4%)	36 (80.0%)	0.346
SARS-CoV-2 virus load [CT value]; mean (±SD)	30.0 (3.46)	21.9 (7.99)	0.024
Laboratory values - of day with highest disease severity*; median (IQR)			
Leukocyte count [1000/µL]	9.90 [6.05;12.8]	13.7 [7.00;24.6]	0.195
- Neutrophile count [1000/µL]	6.62 [4.34;10.5]	8.96 [4.74;12.2]	0.481
- Lymphocyte count [1000/µL]	0.62 [0.42;1.02]	0.64 [0.42;1.47]	0.920
C reactive protein [mg/L]	116 [73.7;177]	154 [106;284]	0.174
Procalcitonin [ng/mL]	0.50 [0.31;1.61]	1.35 [0.37;5.12]	0.121
Interleukin-6 [pg/mL]	134 [87.1;176]	459 [79.9;2805]	0.099
Ferritin [µg/L]	1289 [681;2534]	3421 [892;7799]	0.051

Platelet count [1000/µL]	197 [142;260]	130 [61.0;268]	0.270
Serum creatinine [mg/dL]	1.10 [0.79;1.49]	1.04 [0.72;1.43]	0.778
D-dimers [µg/mL]	2.23 [1.16;4.36]	3.65 [2.14;6.57]	0.125
Total bilirubin [mg/dL]	0.58 [0.32;0.85]	0.80 [0.50;1.32]	0.089
IgM serum concentration [mg/dL]	57.0 [51.0;87.0]	65.0 [45.0;95.0]	0.317
IgA serum concentration [mg/dL]	222 [206;239]	214 [156;258]	0.221
IgG serum concentration [mg/dL]	984 [977;992]	1000 [921;1300]	0.332
**Adjunctive therapies**; n (%)			
Corticosteroids	17 (89.5%)	40 (88.9%)	1.000
Interleukin-6 receptor antagonist	1 (5.26%)	8 (17.8%)	0.260
Remdesivir	2 (10.5%)	8 (17.8%)	0.710

ACEI: Angiotensin converting-enzyme inhibitor; ARB: Angiotensin II receptor blocker; ICU: Intensive care unit; CT: cycle threshold; PaO_2: Partial pressure of oxygen; FiO_2: Fraction of inspired oxygen; PEEP:Positive end expiratory pressure; P_{insp}: Inspiratory plateau pressure; SOFA-Score: Sequential Organ Failure Assessment Score ; AKI: Acute kidney injury; KDIGO: Kidney Disease: Improving Global Outcomes; * Day of the most critical medical condition within the first 10 days after ICU admission.

Table S8: **Baseline characteristics for subgroup without malignancies (n=252).**

	Control group (N=151)	IGAM group (N=101)	p value
**Demographics**			
Age [years], mean (±SD)	61.9 (11.8)	58.8 (12.2)	0.049
Female sex, n (%)	39 (25.8%)	21 (20.8%)	0.442
Body mass index [kg/m^2], mean (±SD)	32.2 (7.18)	30.6 (5.88)	0.047
**Comorbidities, n (%)**			
None	26 (17.2%)	24 (23.8%)	0.265
Hypertension	100 (66.2%)	58 (57.4%)	0.200
Cardiovascular disease	42 (27.8%)	23 (22.8%)	0.453
Chronic heart failure	11 (7.28%)	13 (12.9%)	0.207
Chronic kidney disease	13 (8.61%)	16 (15.8%)	0.118
Chronic obstructive pulmonary disease	8 (5.30%)	11 (10.9%)	0.160
Diabetes mellitus	54 (35.8%)	27 (26.7%)	0.172
Malignant disease	n/a	n/a	-
**Permanent medication, n (%)**			
None	50 (33.1%)	34 (33.7%)	1.000
ACEI	41 (27.2%)	21 (20.8%)	0.318
ARB´s	27 (17.9%)	11 (10.9%)	0.180
Beta blockers	47 (31.1%)	33 (32.7%)	0.904
Platelet aggregation inhibitors	36 (23.8%)	22 (21.8%)	0.820
Anticoagulants	12 (7.95%)	10 (9.90%)	0.756
Corticosteroids	11 (7.28%)	13 (12.9%)	0.207
Immunosuppressive agents	4 (2.65%)	7 (6.93%)	0.123
Polypharmacy (≥ 5 drugs)	59 (39.1%)	31 (30.7%)	0.220

COVID-19 course - day with highest disease severity*			
Days after ICU admission [days], median (IQR)	3.80 (3.50)	4.74 (3.57)	0.040
Respiratory Support			0.145
- Supplemental oxygen, n(%)	2 (1.32%)	1 (0.99%)	
- High-flow oxygen device, n(%)	12 (7.95%)	3 (2.97%)	
- Non-invasive ventilation, n(%)	9 (5.96%)	12 (11.9%)	
- Mechanical ventilation, n(%)	128 (84.8%)	85 (84.2%)	
Horowitz index [PaO2/FiO2], median (IQR)	127 (71.3)	106 (52.2)	0.005
P_{insp} [cmH_2O], median (IQR)	26.5 (5.75)	26.1 (5.52)	0.628
PEEP [cmH2O], median (IQR)	11.8 (3.41)	11.9 (2.97)	0.882
Murray Score; median (IQR)	11.3 (3.05)	12.2 (2.64)	0.022
SOFA Score; median (IQR)	9.47 (3.97)	10.5 (4.00)	0.037
AKI KDIGO stage, n (%)			0.187
-No acute renal injury	88 (58.3%)	51 (50.5%)	
-1	19 (12.6%)	8 (7.92%)	
-2	6 (3.97%)	7 (6.93%)	
-3	38 (25.2%)	35 (34.7%)	
Vasopressor support, n (%)	116 (76.8%)	83 (82.2%)	0.387
SARS-CoV-2 virus load [CT value]; mean (±SD)	30.1 (6.19)	25.6 (6.36)	0.002
Laboratory values - of day with highest disease severity*; median (IQR)			
Leukocyte count [1000/µL]	12.0 [8.70;16.2]	11.7 [8.37;18.2]	0.728
- Neutrophile count [1000/µL]	9.79 [6.84;14.0]	9.32 [6.34;15.1]	0.699
- Lymphocyte count [1000/µL]	0.90 [0.50;1.40]	1.00 [0.58;1.48]	0.762
C reactive protein [mg/L]	150 [85.2;215]	151 [114;243]	0.217
Procalcitonin [ng/mL]	0.51 [0.19;1.04]	0.70 [0.31;2.15]	0.014
Interleukin-6 [pg/mL]	140 [58.9;404]	209 [90.4;509]	0.195
Ferritin [µg/L]	1304 [704;2036]	1628 [973;2626]	0.047
Platelet count [1000/µL]	259 [160;336]	196 [138;290]	0.012
Serum creatinine [mg/dL]	0.92 [0.69;1.61]	1.16 [0.75;1.71]	0.089
D-dimers [µg/mL]	2.47 [1.22;4.66]	2.33 [1.15;8.55]	0.738
Total bilirubin [mg/dL]	0.61 [0.40;1.10]	0.80 [0.51;2.00]	0.016
IgM serum concentration [mg/dL]	78.5 [61.5;101]	94.0 [77.0;131]	0.270
IgA serum concentration [mg/dL]	234 [156;314]	215 [194;482]	0.369
IgG serum concentration [mg/dL]	785 [737;958]	1060 [693;1180]	0.414
**Adjunctive therapies**; n (%)			
Corticosteroids	122 (80.8%)	85 (84.2%)	0.606
Interleukin-6 receptor antagonist	21 (13.9%)	4 (3.96%)	0.018
Remdesivir	34 (22.5%)	14 (13.9%)	0.121

ACEI: Angiotensin converting-enzyme inhibitor; ARB: Angiotensin II receptor blocker; ICU: Intensive care unit; CT: cycle threshold; PaO_2: Partial pressure of oxygen; FiO_2: Fraction of inspired oxygen; PEEP:Positive end expiratory pressure; P_{insp}: Inspiratory plateau pressure; SOFA-Score: Sequential Organ Failure Assessment Score ; AKI: Acute kidney injury; KDIGO: Kidney Disease: Improving Global Outcomes; * Day of the most critical medical condition within the first 10 days after ICU admission.
